# Network Pharmacology- and Molecular Docking-Based Identification of Potential Phytocompounds from *Argyreia capitiformis* in the Treatment of Inflammation

**DOI:** 10.1155/2022/8037488

**Published:** 2022-01-31

**Authors:** Ahmad J. Obaidullah, Mohammed M. Alanazi, Nawaf A. Alsaif, Ashwag S. Alanazi, Hussam Albassam, Alanazi AZ, Osama I. Alwassil, Ali M. Alqahtani, Abu Montakim Tareq

**Affiliations:** ^1^Department of Pharmaceutical Chemistry, College of Pharmacy, King Saud University, P.O. Box 2457, Riyadh 11451, Saudi Arabia; ^2^Department of Pharmaceutical Sciences, College of Pharmacy, Princess Nourah Bint Abdulrahman University, Riyadh 84428, Saudi Arabia; ^3^Department of Pharmacology and Toxicology, College of Pharmacy, King Saud University, P.O. Box 2457, Riyadh 11451, Saudi Arabia; ^4^Department of Pharmaceutical Sciences, College of Pharmacy, King Saud bin Abdulaziz University for Health Sciences, Riyadh 11481, Saudi Arabia; ^5^Department of Pharmacology, College of Pharmacy, King Khalid University, Abha 62529, Saudi Arabia; ^6^Department of Pharmacy, International Islamic University Chittagong, Chittagong 4318, Bangladesh

## Abstract

The methanolic extract of *Argyreia capitiformis* stem was examined for anti-inflammatory activities following network pharmacology analysis and molecular docking study. Based on gas chromatography-mass spectrometry (GC-MS) analysis, 49 compounds were identified from the methanolic extract of *A. capitiformis* stem. A network pharmacology analysis was conducted against the identified compounds, and Kyoto Encyclopedia of Genes and Genomes (KEGG) pathway analysis and Gene Ontology analysis of biological processes and molecular functions were performed. Six proteins (IL1R1, IRAK4, MYD88, TIRAP, TLR4, and TRAF6) were identified from the KEGG pathway analysis and subjected to molecular docking study. Additionally, six best ligand efficiency compounds and positive control (aspirin) from each protein were evaluated for their stability using the molecular dynamics simulation study. Our study suggested that IL1R1, IRAK4, MYD88, TIRAP, TLR4, and TRAF6 proteins may be targeted by compounds in the methanolic extract of *A. capitiformis* stem to provide anti-inflammatory effects.

## 1. Introduction

Inflammation describes a biological process that occurs in tissues to protect the host against harmful stimuli, such as microorganisms and abnormal or damaged cells. Inflammation stimulates the immune system and regulates protective responses via immune cells, blood vessels, and molecular biological agents [1, 2]. Many chronic diseases, including cardiovascular and gastrointestinal illnesses, diabetes, rheumatism, and cancer, are associated with upregulated inflammation [3]. Chronic diseases represent a major human health concern according to the World Health Organization (WHO). The incidence of chronic inflammation-related disorders is expected to steadily increase in the United States (US) over the next 30 years. Approximately 125 million people in the US were diagnosed with chronic diseases in 2000, with 61 million (21%) having multiple conditions [4–6]. Typically, cellular and molecular mechanisms and interactions among various factors can efficiently limit the potential damage and prevent further infection during an acute inflammatory response, resulting in the eventual repair of cellular homeostasis following the resolution of acute inflammation. However, uncontrolled acute inflammation can develop into chronic inflammation, causing a range of chronic inflammatory diseases [7]. Three out of five people worldwide die from chronic inflammatory conditions, such as stroke, respiratory infections, cardiovascular diseases, cancers, diabetes, and obesity [4–6]. A growing interest in the use of medicinal plants has developed for the treatment and management of diseases in an effort to identify safer and more efficient anti-inflammatory agents for the prevention of inflammatory conditions rather than using synthetic anti-inflammatory drugs [8].

*Argyreia capitiformis* (Poir.) Ooststr. is a member of the Convolvulaceae family of the *Argyreia* genus, which is not toxic and has medicinal and ornamental uses [9, 10]. Traditionally, a paste made from the leaves of *A. capitiformis* has been used as an effective treatment of bruising on the legs. *A. capitiformis* has also been used in traditional medicine as a purgative and to treat sexual debility and ear pain [10–12]. Several studies have been reported for the *Argyreia* species, including antioxidant, anti-inflammatory, immunomodulatory, and CNS activities with several bioactive compounds [13–15]. However, no such study has been evaluated for *A. capitiformis* except the recent study on the methanolic extract of *A. capitiformis* leaves that suppressed the nuclear factor-kappa B (NF-*κ*B) pathway and inhibited the lipopolysaccharide-induced production of nitric oxide and inducible nitric oxide synthase in RAW 264.7 cells, demonstrating anti-inflammatory activities [9]. However, the chemical compounds found in *A. capitiformis* that are responsible for these anti-inflammatory effects and the underlying mechanisms remain unknown. Further research is necessary to identify the potential lead compounds responsible for these biological anti-inflammatory outcomes.

Network pharmacology has become a widely accessible analysis method following the increased availability of biomedical data sets during the postgenomic period, supporting the growth of the fields of systems biology and polypharmacology [16]. Complex compound-gene and compound-protein interactions can be evaluated systematically to develop a prototype for efficient therapy. New therapeutic mechanisms may be discovered by network pharmacology analysis, which is oriented toward a “multi-goals, multi-disease” paradigm rather than “one target, one drug” [17–19]. Network pharmacology represents an effective method for selecting and elucidating the synergistic effects among bioactive chemicals through the mechanistic exploration of effects on multiple disease pathways [19, 20]. Additionally, spectrometric and chromatographic technologies used in the initial evaluation of medicinal plants provide valuable information on bioactive activities that aid in the selection of biologically active species. Alkaloids, phenolic compounds, organic acids, esters, and amino acids are among the chemicals that GC-MS can detect quickly and accurately. Thus, in this investigation, GC-MS was used to detect and identify phytochemical constituents in *A. capitiformis* [21–24].

The network pharmacology approach connects targeted genes with the effects of bioactive compounds; thus, the present study was designed to elucidate the anti-inflammatory effects of the methanolic extract of *A. capitiformis* stem using a network pharmacology approach. Bioactive compounds in the methanolic extract of *A. capitiformis* stem were identified for this study using gas chromatography-mass spectrometry (GC-MS) analysis, followed by a molecular docking assay to investigate potential ligand-receptor interactions, including the assessment of binding affinity and stability.

## 2. Materials and Methods

### 2.1. Plant Extraction

The stems of *A. capitiformis* were collected from the Sitakunda Eco-park, Chittagong, Bangladesh, in March 2020 and later identified by a taxonomist. The stems were subjected to air-drying and ground to a coarse powder. The powder (200 g) was soaked in methanol (1 L) for 7 days [25, 26]. Subsequently, the extract was filtered through Whatman filter paper and evaporated at 45°C. After the evaporation, 2.67 g of the black methanol extract yield (1.34%) was collected in an amber glass vial and refrigerated at 4°C until further use.

### 2.2. GC-MS Analysis

An Agilent GC 7890A (Agilent Technologies Inc., Wilmington, DE, USA), combined with a triple-axis detector 5975 C single-quadrupole mass spectrometer, was used for GC-MS analysis. The chromatographic column was an Agilent HP 5MS column (30 m × 0.25 mm × 0.25 *µ*m film thickness), using high-purity helium as the gas carrier at a flow rate of 1 mL per min. The injector temperature was 230°C, and the sample was injected using a splitless injector at 20 : 1. The temperature was set primarily to 40°C (held for 1 min), raised to 150°C at a rate of 5°C per min (held for 2 min), before being increased to 300°C at a rate of 5°C per min (held for 10 min). The temperature of the MS ion source was set to 150°C, and the temperature of the inlet line was set to 280°C. The scan range was set between 50 and 550 mass, with 70 eV electron energy and a 4-min solvent delay. Finally, by comparing the spectra against the NIST 2008 database (National Institute of Standard and Technology library), tentative compounds were identified. The total analysis time required for the sample was 65 min [27].

### 2.3. Network Pharmacology

The network pharmacology analysis was performed using the STITCH platform (http://stitch.embl.de/) to identify putative associations between the identified compounds and target genes. Multiple compound targets were identified using the *Homo sapiens* genome [27–29]. Multiple functional nodes and edges were identified in the network. Kyoto Encyclopedia of Genes and Genomes (KEGG) analysis was performed on the components identified in the network to obtain a biological interpretation of the vast list of potential targets and to identify potential anti-inflammatory pathways that are targeted.

The STRING (search tool for retrieval of interacting genes) database (https://string-db.org), which includes predicted protein-protein interactions (PPIs), was used to predict functional protein interactions [30].

### 2.4. Molecular Docking Study

#### 2.4.1. Protein Selection and Preparation

Six targeted proteins were selected from the KEGG analysis: interleukin 1 (IL-1) receptor type 1 (IL1R1; PDB: 1ITB) [31], IL-1 receptor-associated kinase 4 (IRAK4; PDB: 6EGA) [32], myeloid differentiation factor 88 (MYD88; PDB: 4EO7) [33], TIR domain-containing adaptor protein (TIRAP; PDB: 4FZ5) [34], Toll-like receptor 4 (TLR4; PDB: 3FXI) [35], and tumor necrosis factor (TNF) receptor-associated factor 6 (TRAF6; PDB: 3HCT) [36]. Protein structures were retrieved in *.pdb* format from the Protein Data Bank (http://www.rcsb.org/pdb). The proteins were prepared using Schrödinger (Maestro v11.1), utilizing the force field OPLS3 [27].

#### 2.4.2. Ligand Preparation

We selected 47 compounds identified from *A. capitiformis,* according to the qualitative GC-MS analysis, which we submitted to the molecular docking study. The selected compounds were retrieved in *.sdf* format from the PubChem database. In addition, aspirin (CID: 2244) was utilized in this study as a positive anti-inflammatory control. The three-dimensional (3D) structures of the selected compounds were constructed in Schrödinger using LigPrep (Maestro v11.1), utilizing the force field OPLS3.

#### 2.4.3. Grid Generation and Molecular Docking

To create receptor grids and execute a molecular docking analysis, Glide (Schrödinger, Maestro v11.1) was used. The grids were generated in Glide with the default settings and the OPLS3 force field. A cubic box with a boundary box (14 Å × 14 Å × 14 Å) was specified for the receptors. All docking studies were conducted using Glide's standard precision (SP) and flexible docking modes, and the lowest docking score for each ligand was recorded.

#### 2.4.4. MM-GBSA and Ligand Efficiency Analysis

The free energies of binding (Δ*G*; kcal/mol) for each ligand and the target receptors were calculated using the Schrödinger software package Prime/MM-GBSA module (OPLS3) [37, 38]. The ligand efficiency (LE) was assessed for each ligand by obtaining the ratio of Δ*G* to the number of heavy atoms (NHA): LE = −(Δ*G*)/NHA [39].

### 2.5. Molecular Dynamics Simulation

The molecular dynamics simulation study was conducted in YASARA dynamics by the aid of AMBER14 force field [40, 41]. The docked complexes were optimized and cleaned, and hydrogen bond network system was oriented. The cubic simulation cell was created where the TIP3P solvation model was used with periodic boundary conditions [42]. The simulation system was neutralized at 310 K temperature, pH 7.4, and 0.9% NaCl. The initial energy minimization was conducted by steepest grained algorithms by simulating annealing methods. The long-range electrostatic interactions were calculated by the particle mesh Ewald (PME) methods with a cutoff radius of 8.0 Å [43, 44]. The simulation time step was set as 2.0 fs. The simulation trajectories were saved after 100 ps and finally run for 20 ns by following the constant pressure and Berendsen thermostat [45]. The simulation trajectories were used to calculate the root-mean-square deviation, solvent accessible surface area, radius of gyration, and hydrogen bond [46–54].

## 3. Results

### 3.1. GC-MS Analysis

In this work, methanol was utilized as the solvent for extraction, resulting in a 1.34% yield. The GC-MS analysis of the *A. capitiformis* stem methanolic extract revealed 49 compounds with different retention times and peak areas (Table 1 and Figure S1). The methanolic extract contained the following identified compounds: stigmast-4-en-3-one (20.78%, RT: 58.161); hexadeca-2,6,10,14-tetraen-1-ol, 3,7,11,16-tetramethyl-, (E,E,E)- (18.36%, RT: 51.477); ursa-9(11),12-dien-3-one (10.35%, RT: 58.55); ursa-9(11),12-dien-3-ol (6.89%, RT: 55.077); 2-(2-hydroxy-2-phenylethyl)-3,5,6-trimethylpyrazine (5.21%, RT: 22.438); longipinane, (E)- (3.86%, RT: 61.245); urs-12-ene (2.95%, RT: 57.274); 2-hydrazino-8-hydroxy-4-phenylquinoline (2.29%, RT: 50.688); and friedelin (2.01%, RT: 59.511).

### 3.2. Network Construction and Biological Process Analysis

A KEGG pathway analysis of potential target genes (IL1R1, IRAK4, MYD88, TIRAP, TLR4, and TRAF6) revealed signaling pathways related with anti-inflammatory effects (see Table 2).  Figure S2 shows the PPI network with 12 proteins (IL1R1, IRAK2, MYD88, IRAK4, IRAK2, MYD88, TIRAP, TRAF6, TLR3, TLR4, TLR5, and TLR6), all of which have anti-inflammatory effects. Tables S1 and S2 represent the biological processes and molecular functions of the genes interacting with the compounds, respectively. Protein-protein interaction (PPI) status of 10 proteins with coexpression is demonstrated in Table S3.

### 3.3. Molecular Docking and Simulation

**Figure 1:**
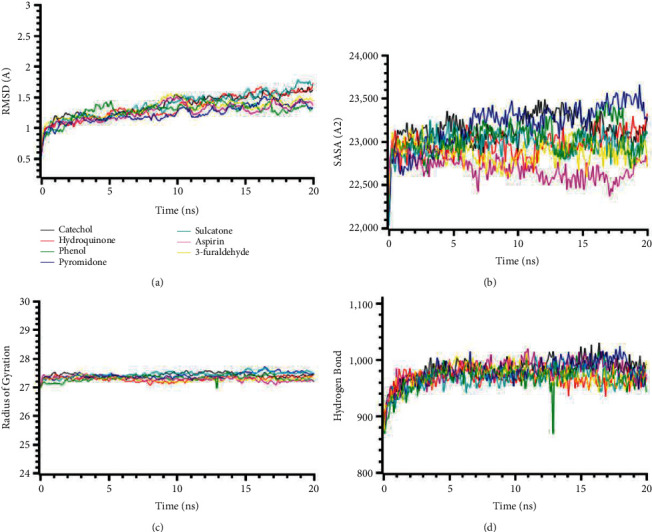
Molecular dynamics simulation of the best ligand efficiencies for compounds against IL1R1 (PDB: 1ITB). (a) Root-mean-square deviation of the complexes (RMSD). (b) Solvent accessible surface area (SASA). (c) Radius of gyration (Rg). (d) Hydrogen bond analysis from the simulation system.

**Figure 2:**
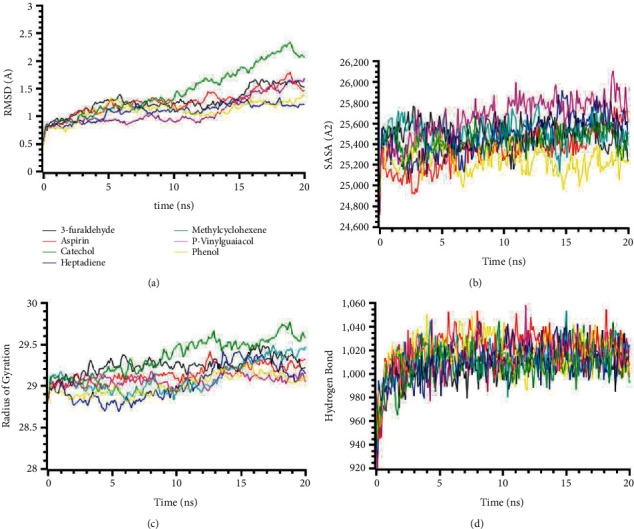
Two-dimensional representations of the best ligand efficiencies for compounds against IRAK4 (PDB: 6EGA). (a) Root-mean-square deviation of the complexes (RMSD). (b) Solvent accessible surface area (SASA). (c) Radius of gyration (Rg). (d) Hydrogen bond analysis from the simulation system.

**Figure 3:**
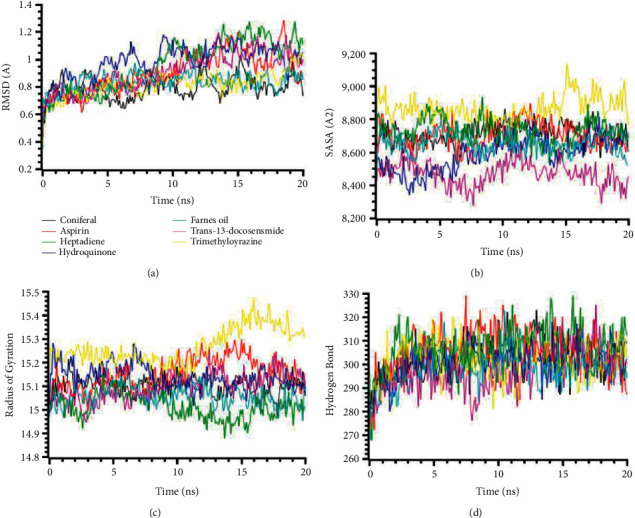
Two-dimensional representations of the best ligand efficiencies for compounds against MYD88 (4EO7). (a) Root-mean-square deviation of the complexes (RMSD). (b) Solvent accessible surface area (SASA). (c) Radius of gyration (Rg). (d) Hydrogen bond analysis from the simulation system.

**Figure 4:**
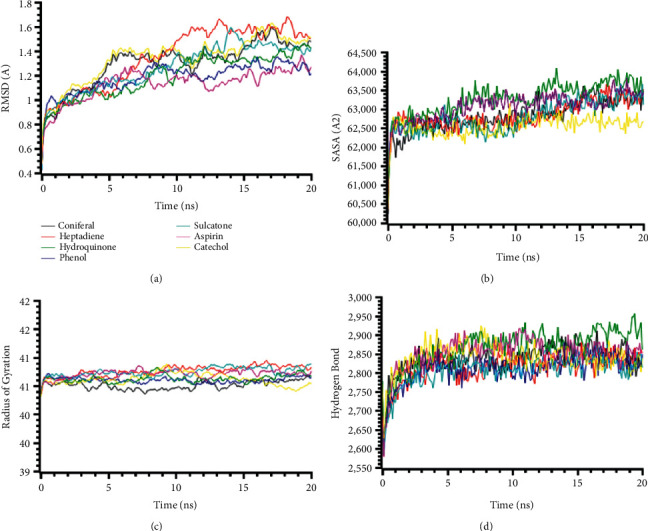
Two-dimensional representations of the best ligand efficiencies for compounds against TIRAP (PDB: 4FZ5). (a) Root-mean-square deviation of the complexes (RMSD). (b) Solvent accessible surface area (SASA). (c) Radius of gyration (Rg). (d) Hydrogen bond analysis from the simulation system.

**Figure 5:**
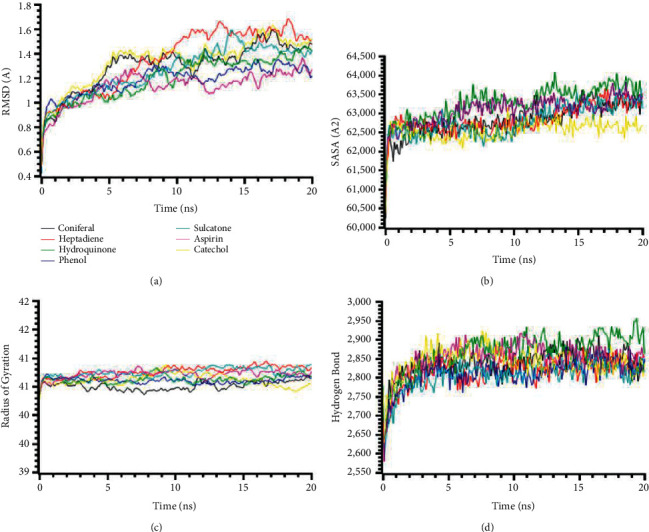
Two-dimensional representations of the best ligand efficiencies for compounds against TLR4 (PDB: 3FXI). (a) Root-mean-square deviation of the complexes (RMSD). (b) Solvent accessible surface area (SASA). (c) Radius of gyration (Rg). (d) Hydrogen bond analysis from the simulation system.

**Figure 6:**
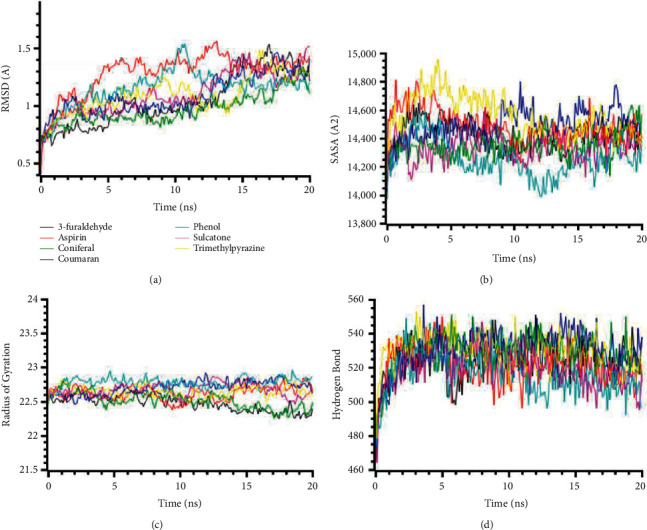
Two-dimensional representations of the best ligand efficiencies for compounds against TRAF6 (PDB: 3HCT). (a) Root-mean-square deviation of the complexes (RMSD). (b) Solvent accessible surface area (SASA). (c) Radius of gyration (Rg). (d) Hydrogen bond analysis from the simulation system.

A total of 47 compounds docking results are presented in Tables 3–8, which show the findings. Aspirin has been used as a positive control for this study. This study's findings reveal that binding energies of most of the ligands to receptors are negative, as later validated by MM-GBSA analysis. The proteins and ligands' molecular interaction is presented in Supplementary Materials (Tables S4–S9). To better understand the docking score, we have studied the ligand efficiency, which demonstrated excellent support for molecular docking scores. The molecular dynamics simulation of the targeted receptors (IL1R1, IRAK4, MYD88, TIRAP, TLR4, and TRAF6) against the best stable compounds is presented in Figures 1–6.

## 4. Discussion

Plants have been used throughout the history of traditional medicine to induce a variety of biological effects, and extensive pharmaceutical resources have recently been devoted to the identification and investigation of new remedies, including those derived from plants. A critical issue encountered by researchers who perform phytoscience is that a single plant can harbor a wide range of bioactive chemicals [55, 56]. The pharmaceutical industry relies on phytochemicals to develop new drugs and therapeutic agents. Finding natural bioactive components is the first step in developing novel drugs. Screening plant extracts for therapeutically active chemicals is a novel strategy. It is important to know that plants have a lot of different types of phytochemicals, which have a lot of different biological properties. These include antioxidant, anti-inflammatory, antidiarrhea, antiulcer, and anticancer activities [57–59]. Determining which compounds are responsible for the biological activities associated with plant materials can help understand toxicities, determine suitable doses, and identify ideal methods for compound extraction. The successful acquisition of components from plant materials depends primarily on the solvent used during the extraction process [60, 61]. The stem methanolic extract was analyzed by GC-MS analysis, and the results indicated 49 different chemicals with varying retention times and peak areas.

**Figure 7:**
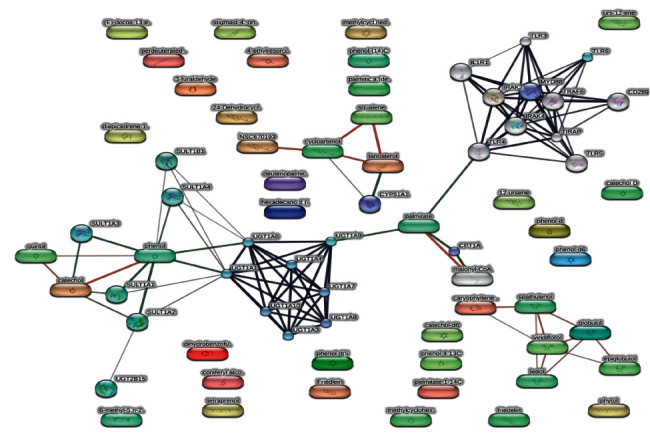
Network pharmacology presentation of phytoconstituents, targets, and pathways.

The network pharmacology analysis was performed to evaluate potential interactions between the identified chemical compounds and proteins, followed by multiple comparisons to determine the number of genes responsible for the anti-inflammatory effects, which was represented by the STITCH platform (Figure 7). Table 2 shows the results of KEGG pathway analysis performed on potential target genes, which identified signaling pathways associated with anti-inflammatory actions. Analyses of biological processes and molecular functions, which identified proteins and pathways with significant values, were also performed (Table S1). A comparison of the compound-gene interaction network and the protein-protein interaction network was performed to reveal biological and molecular functions (Table S2). From a molecular and functional perspective, these findings can assist in understanding the computational rules of compounds that have the potential to treat diseases. For example, protein kinase C (PKC) binding is known to treat inflammatory diseases [62], and the present results showed that binding with TIRAP, UGT1A10, and UGT1A7 was significant (*p* = 0.0447) and similar to that for PKC. Also, retinoic acid binding has a great impact on the immune system and the inflammatory response. Our findings identified the genes *UGT1A1, UGT1A3, UGT1A7, UGT1A8*, and *UGT1A9* as being the most significant interactions for the compounds in our extract (*p* = 2.69*E* − 06).

As shown in Table 2, KEGG pathway analysis for inflammatory responses identified *IL1R1, IRAK4, MYD88, TIRAP, TLR4,* and *TRAF6* in the NF-*κ*B signaling pathway, which has a *p*-value of 1.35*E* − 05. Similar results were demonstrated for the inflammatory response in the biological process analysis, which identified the MYD88-dependent TLR signaling pathway (*p* = 2.19*E* − 07; *IRAK2, IRAK4, MYD88, TIRAP, TLR4, TLR5, TLR6,* and *TRAF6*), the TLR4 signaling pathway (*p* = 9.14*E* − 07; *IRAK2, IRAK4, MYD88, TIRAP, TLR3, TLR4, TLR6,* and *TRAF6*), the TLR1:TLR2 signaling pathway (*p* = 2.31*E* − 06; *IRAK2, IRAK4, MYD88, TIRAP, TLR4, TLR6,* and *TRAF6*), the TLR 2 pathway (*p* = 2.54*E* − 06; *IRAK2, IRAK4, MYD88, TIRAP, TLR4, TLR6,* and *TRAF6*), the TLR signaling pathway (*p* = 3.15*E* − 06; *IRAK2, IRAK4, MYD88, TIRAP, TLR3, TLR4, TLR5,* and *TRAF6*), positive regulation of interleukin-6 production (*p* = 2.06*E* − 05; *MYD88, TIRAP, TLR3, TLR4, TLR6,* and *TRAF6*), NF-*κ*B signaling (*p* = 0.000393; *IRAK2, TIRAP, TLR3, TLR4,* and *TRAF6*), NF-*κ*B signaling (*p* = 0.000601; *IRAK4, MYD88, TIRAP, TLR3, TLR4, TLR6,* and *TRAF6*), TLR5 signaling pathway (*p* = 0.000712; *IRAK2, IRAK4, MYD88, TLR5,* and *TRAF6*), and TLR10 signaling pathway (*p* = 0.000712; *IRAK2, IRAK4, MYD88, TLR5, TRAF6*). Six genes were identified as being associated with the NF-*κ*B signaling pathway, suggesting anti-inflammatory effects, in support of a previous study that identified the inhibition of NF-*κ*B functional pathways in RAW 264.7 cells following treatment with a methanolic extract of *A. capitiformis* leaves [9].

Although a remarkable amount of functional and structural data has been compiled for each identified protein, our knowledge regarding protein-protein relationships remain scattered. The purpose of the STRING database is the collection, scoring, integration, and complementation with computational predictions for all public sources of PPIs [63–66]. In the present study, 10 proteins (*IL1R1, IRAK4, IRAK2, MYD88, TIRAP, TRAF6, TLR3, TLR4, TLR5,* and *TLR6*) were used to analyze a PPI network (Figure S2), which were all significantly (*p* < 1.0*e* − 16) related to anti-inflammatory functions. Signals from the various TLRs are diverse, but all TLRs are activated by the binding of TIR-containing adaptor proteins (e.g., TIRAP activates MYD88, and TRAM activates TRIF). IRAK4, IRAK1, IRAK2, and TRAF6 are all activated by MYD88. MYD88 binding results in the phosphorylation and release of IRAK1 from the cell membrane into the cytoplasm, where it interacts with and activates the transforming growth factor-*β*-activated kinase 1 (TAK1) downstream. TAK1 activates the IKK*β*-to-I*κ*B-*α*-to-NF-*κ*B pathway, inducing the transcription of proinflammatory genes. TAK1 also influences gene expression by activating mitogen-activated protein kinase (MAPK) cascades. TRAF6, *β*RIP1, and TAK1 are activated by the binding of TLR with TRIF, resulting in the activation of MAPK, interferon regulatory factor 3 (IRF3), NF-*κ*B, and interferon- transcription activation. The TRIF pathway also promotes the release of proinflammatory cytokines, although to a lesser degree than the MYD88 pathway. TLRs are a class of specific receptors that are key players in mounting an effective innate immune response to infection [67–69].

Subsequently, gene co-occurrence can be used to identify gene families whose patterns exhibit similarity across genomes. Three types of analyses have been used to examine genomes (neighborhood, fusion, gene co-occurrence) based on the systemic comparison of all-against-all genomes to evaluate the impacts of historical genome restructurings, genetic gains and losses, and gene fusion [70, 71]. In the present study, 100% sequence conservation was observed for the ten proteins (IL1R1, IRAK4, IRAK2, MYD88, TIRAP, TRAF6, TLR3, TLR4, TLR5, and TLR6) selected for the PPI network analysis, as shown in Figure S3. Additionally, gene coexpression was also studied in the present study, as summarized in Table S3 and Figure S4. The coexpression pathway is predicated by performing gene-by-gene correlation testing across many gene expression databases. STRING reconstructs and maps this enormous series of experiments, which is stored on the NCBI database along with transcript data [63, 72]. The present findings suggested co-expression among the ten selected proteins (IL1R1, IRAK4, IRAK2, MYD88, TIRAP, TRAF6, TLR3, TLR4, TLR5, and TLR6) in *Homo sapiens*. In addition, coexpression (transferred) of three more genes was observed in *Gallus gallus*, *Mus musculus*, and *Rattus norvegicus*.

Structure-based drug discovery is gradually becoming a key technique for facilitating the rapid and cost-effective discovery and optimization of lead compounds. The use of a rational, structure-based drug design strategy is more efficient than conventional drug development techniques because this approach seeks to understand the molecular basis of diseases and incorporates information regarding the biological target's 3D structure during the drug design process [37]. A molecular docking study was incorporated into the present study to predict the complex structure formed by ligand-protein binding and analyze the ligand's conformational space within the protein-binding site. A score function is then used for each docking analysis to assess the free energy of the interaction between the protein and ligand [37, 73, 74]. Additionally, the LE is calculated, which can enrich docking functions and allow for the coordination between docking outcomes and experimental results. Critical information regarding a molecule's properties, such as the NHA, can then be combined into a single table [39].

IL-1 controls a range of innate immune pathways, making it a key regulator of inflammation [75]. Two IL-1 cell surface receptors and a decoy receptor have been identified, including IL1R1 and IL1R2. First, IL-1 binds with IL1R1, inducing the formation of a heterodimer between IL1R1 and either IL-1RAcP or IL1R3, followed by IL-1 receptor-associated kinase (IRAK) and MyD88. The inflammatory response induced by IL1R1 occurs when IL1R1 binds with either the IL-1*α* or IL-1*β* ligands, whereas the T-lymphocytes, fibroblastic cells, epithelial cells, and endothelial cells have been indicated [76–78]. In the present study, selected six target proteins based on the network pharmacology analysis for the molecular docking study: IL1R1 (PDB: 1ITB), IRAK4 (PDB: 6EGA), and MYD88 (PDB: 4EO7) was used for the docking study. For IL1R1 (PDB: 1ITB), the majority of the 47 tested compounds demonstrated good docking scores, except for 12 compounds (Table 3). Among the 33 compounds with good docking scores, catechol (3.02), phenol (2.94), 4(1H)-pyrimidinone, 6-hydroxy- (2.79), hydroquinone (2.70), 3-furaldehyde (2.51), and sulcatone (2.51) showed the best ligand efficiencies, compared with the LE value of 1.63 for the positive control aspirin (Table S4 and Figure S5). The compounds with the best ligand efficiencies were found to interact with LYS-93 by H-bond (distances >4 Å), and catechol interacted via two H-bonds. Similar findings were also observed for the positive control aspirin. According to an earlier study, the noncontiguous binding epitope containing LYS-93 was identified for IL-1*β* [79].

IRAK4 (PDB: 6EGA) also exhibited similar findings in the docking experiment, with only 12 compounds that did not display any interactions. Catechol (4.45), phenol (4.35), p-vinylguaiacol (4.15), methylcyclohexane (4.08), 3-furaldehyde (4.06), and 4-methyl-1,5-heptadiene (3.94) showed the best LE values, as shown in Table 4. The LE value of aspirin was 2.39. The molecular interactions between the compounds with the best LE values and IRAK4 (PDB: 6EGA) are presented in Table S5 and Figure S6. ASP-329 formed H-bonds with catechol (4.02, 3.48 Å), p-vinylguaiacol (4.05, 4.06 Å), phenol (3.49 Å), and aspirin (4.24 Å), whereas no H-bond interactions were observed for 4-methyl-1,5-heptadiene or methylcyclohexane. Additionally, 3-furaldehyde interacted with MET-265 (4.0 Å) via one H-bond. Remarkably, ASP-329, MET-265, and LYS-213 were all reported to interact with IRAK4 in a previous study [32].

Additionally, MYD88 (PDB: 4EO7) was also studied as a potential target of the anti-inflammatory effects of the methanolic extract of *A. capitiformis* stems. In the molecular docking experiment, 29 compounds were found to interact with MYD88 (Table 5). The best LE values for MYD88 (PDB: 4EO7) were identified for 4-methyl-1,5-heptadiene (1.84), 2-(2-hydroxy-2-phenylethyl)-3,5,6-trimethylpyrazine (1.81), trans-13-docosenamide (1.75), coniferol (1.73), (Z, E)-farnesol (1.65), and hydroquinone (1.55). The positive control aspirin had an LE value of 0.45, which was lower than the LE values for 24 of the identified compounds. The molecular interactions between the compounds with the best LE values and MYD88 (PDB: 4EO7) are presented in Table S6 and Figure S7. ASP-156 was reported to form H-bonds with coniferol, 2-(2-hydroxy-2-phenylethyl)-3,5,6-trimethylpyrazine, and (Z,E)-farnesol, whereas hydroquinone interacted with ASP-156 through the formation of hydrophobic bonds (3.51 Å). No H-bond interactions were reported for 4-methyl-1,5-heptadiene. The other compound, trans-13-docosenamide, interacted with ARG-160 (5.21 Å) and GLU-159 (4.36 Å) via H-bonds.

TIRAP, also known as MYD88 adapter-like (Mal), is an important link between MYD88 and the receptor complex formed by TLR2 and TLR4 activation following bacterial infection [32]. MYD88 activates IRAK1 and IRAK4 and eventually activates TRAF6, causing the prototypic inflammatory transcription factor NF-*κ*B to translocate to the nucleus [80, 81]. TIRAP is the second adaptor that has been identified to mediate NF-*κ*B activation through the activation of TLR4 and TLR2 signaling pathways [82–85]. The molecular docking of TIRAP (PDB: 4FZ5) was examined against the 45 compounds identified in the methanolic extract of *A. capitiformis* stems, of which 35 demonstrated interactions and 10 did not (Table 6). The best interacting compounds were phenol (3.06), 4-ethylresorcinol (2.96), catechol (2.84), methylcyclohexane (2.72), hydroquinone (2.71), and 3-furaldehyde (2.50), compared with the positive control aspirin, which exhibited an LE of 1.82 (Figure S8). The molecular interactions between the compounds with the best LE values and TIRAP (PDB : 4FZ5) are presented in Table S7. LEU-107 formed H-bond with 3-furaldehyde, catechol, phenol, and aspirin, and a hydrophobic interaction was also observed for catechol, methylcyclohexane, and phenol. Another protein residue, LYS-210, interacted with 3-furaldehyde, 4-ethylresorcinol, hydroquinone, and phenol via hydrophobic interactions. In a previous study, LYS-210 was reported to form a hydrophobic interaction with NF-*κ*B [86].

In addition, TLR4 (PDB: 3FXI) was compared against 45 compounds found in the methanolic extract of *A. capitiformis* stems, although 17 compounds did not interact with TLR4 (Table 7). Phenol (4.59), 4-methyl-1,5-heptadiene (4.45), catechol (4.39), sulcatone (4.17), hydroquinone (4.01), and coniferol (4.003) displayed the highest LE values, whereas aspirin showed an LE value of 2.75. The molecular interactions between the compounds with the best LE values and TLR4 (PDB: 3FXI) are presented in Table S8 and Figure S9. SER-441 was found to form H-bond interactions with phenol and sulcatone, whereas hydrophobic interactions were exhibited for phenol, hydroquinone, catechol, and coniferol. VAL-82 interacted via hydrophobic interactions with coniferol, 4-methyl-1,5-heptadiene, catechol, phenol, and hydroquinone. In a previous study, SER-441 was reported to interact strongly with apigenin-7-O-glucoside [87]. Similar interactions were also observed for ILE-80 and VAL-82 [88].

Finally, TRAF6 (PDB: 3HCT) was assessed against the 45 chemicals found in the methanolic extract of *A. capitiformis* stems, of which 34 were identified as interacting (Table 8). Sulcatone (2.47), phenol (2.44), coniferol (2.44), 3-furaldehyde (2.41), 2-(2-hydroxy-2-phenylethyl)-3,5,6-trimethylpyrazine (2.33), and coumaran (2.32) displayed the highest LE values compared with aspirin (2.07). The molecular interactions between the compounds with the best LE values and TRAF6 (PDB: 3HCT) are presented in Table S9 and Figure S10. ARG-6 formed H-bond interactions with coniferol, 2-(2-hydroxy-2-phenylethyl)-3,5,6-trimethylpyrazine, and aspirin, whereas hydrophobic interactions were identified for 2-(2-hydroxy-2-phenylethyl)-3,5,6-trimethylpyrazine and sulcatone. GLN-54 also formed H-bond interactions with multiple compounds, including 2-(2-hydroxy-2-phenylethyl)-3,5,6-trimethylpyrazine and 3-furaldehyde.

The root-mean-square deviations from the C-alpha atoms from the docked complexes IL1R1 (PDB: 1ITB) proteins are illustrated in Figure 1. The complexes' RMSD values were calculated to find out the deviations among the simulation complexes and structural stability.  Figure 1(a) demonstrates that the complexes had similar RMSD profiles and did not fluctuate much in the simulation trajectories. The RMSD profile of the complexes reached the steady state after 5 ns and maintained the structural stability till the last periods of the simulations, which defines the stability of the complexes. The SASA of the complexes was analyzed to find out the change in the surface area. The higher SASA represents the expansion of the surface area of the protein, whereas the lower SASA indicates the truncated nature of the complexes.  Figure 1(b) shows that the complexes were in a stable state in SASA. The radius of gyration defines the labiality and mobility of the complexes, where Figure 1(c) indicates the lower deviations. The hydrogen bond patterning follows a similar stable profile (Figure 1(d)).

The molecular dynamics simulation of IRAK4 (PDB: 6EGA) is presented in Figure 2. The RMSD of the complexes had a stable trend in the RMSD profile for all the complexes except catechol. The higher RMSD of these complexes defines the higher flexibility of these compounds in the simulating environments (Figure 2(a)). The SASA profile of the complexes was stable, did not fluctuate much, and had a steady trend in SASA (Figure 2(b)). This SASA profile correlates with the stable and rigid profile of the complexes (Figure 2(c)). The radius of gyration and hydrogen bond pattern systems were similar and did not change too much in the simulations (Figure 2(d)).

The RMSD of the MYD88 (4EO7) protein complexes had a lower level of fluctuations, and lower deviations were observed across the compounds. All compounds had a lower RMSD than 2.5 Å in whole simulation periods (Figure 3(a)). The SASA of the MYD88 (4EO7) complexes were stable, but the complex of trans-13-docosenamide had a lower SASA than the other complexes. This SASA profile of trans-13-docosenamide defines the MYD88 (4EO7) experienced the condensed conformation upon binding with the corresponding ligands (Figure 3(b)). The 2-(2-Hydroxy-2-phenylethyl)-3,5,6-trimethylpyrazine complexes had a higher Rg than other complexes, which defines the complexes' flexible nature than other complexes (Figure 3(c)). These complexes also had a higher SASA in Figure 3(b), which depicts the changes in the confirmations than other complexes in simulated environments. The hydrogen-bonding pattern of the complexes for MYD88 (4EO7) protein was found stable and did not change too much in simulations (Figure 3(d)).

The RMSD profile of TIRAP (PDB: 4FZ5) complexes is illustrated in Figure 4. All complexes from TIRAP (PDB: 4FZ5) protein had an initial rise of RMSD due to a higher degree of flexibility but stabilized subsequently after 5 ns times. The complex 4-ethylresorcinol had a higher RMSD than the other complexes, which might be responsible for the more remarkable conformational changes and the flexibility of the compounds (Figure 4(a)). The SASA of the complexes had a stable and similar trend for all the compounds. But the complex phenol had a lower SASA profile at the last phase of SASA, which defines the complexes' truncated nature in simulations (Figure 4(b)). The radius of gyration profile of the complexes had a lower trend, which correlates with the less flexibility of the complexes (Figure 4(c)). The hydrogen bond patterning of the complexes had a stable profile in Figure 4(d).

The molecular dynamics simulation study of the TLR4 (PDB: 3FXI) and complexes was done to analyze the structural deviation in the docked structure. The root-mean-square deviations of all complexes are illustrated in Figure 5(a). The RMSD value of the complexes initially followed the upper trend from the beginning. This might be happening due to the higher flexibility level. But all the complexes from TLR4 (PDB: 3FXI) had a stable profile after 10 ns times and followed a similar trend until the last periods, demonstrating structural stability. The SASA of the TLR4 (PDB: 3FXI) complexes had lowered the degree of the deviations from the beginning and followed lower fluctuations, which define no changes in the surface area of the complexes (Figure 5(b)). The Rg and hydrogen bond patterns of the simulation systems were stable and did not change too much, which correlates with the structural stability (Figures 5(c) and 5(d)).

The docked complexes from the TRAF6 (PDB: 3HCT) protein and their simulation descriptors are illustrated in Figure 6. The RMSD profile of the TRAF6 (PDB: 3HCT) complexes defines that the phenol and aspirin had a higher level of RMSD than other complexes, which correlates with the comparative higher degree of the deviations of the complexes. All complexes had reached a stable state after 5 ns of the simulation times. Moreover, the complexes exhibit RMSD lower than 2.5 Å, which defines the complexes with a higher rigidity degree (Figure 6(a)). The SASA profile of the complexes had a lower deviation, as illustrated in Figure 6(b). The phenol had a lower SASA value than all the compounds, indicating the truncated nature of the protein complexes compared with the others. Moreover, the radius of gyration from Figure 6(c) demonstrates that the complexes had a lower degree of deviations, and no significant higher fluctuations were observed. This Rg profile indicates the complexes had lower mobility and flexibility during the simulation times. The hydrogen bond pattern of the complexes was stable during the simulations (Figure 6(d)).

## 5. Conclusions

The network pharmacology analysis revealed key pathways involved in the anti-inflammatory activities induced by the chemical compounds found in the methanolic extract of *A. capitiformis* stem. Six inflammation pathways were obtained from the KEGG pathway analysis (*IL1R1, IRAK4, MYD88, TIRAP, TLR4,* and *TRAF6*), and molecular docking studies of these pathways revealed that the identified chemical compounds had strong binding affinities with these pathway components. The current study discovered that 3-furaldehyde, phenol, catechol, and hydroquinone were effective anti-inflammatory compounds found within the methanolic extract of *A. capitiformis* and played an important part in the inflammation pathway by targeting these six proteins. Additional *in vitro* and *in vivo* experiments will be helpful to validate and optimize the findings of this study.

## Figures and Tables

**Table 1:** GC-MS analysis of the methanolic extract of *Argyreia capitiformis* stem.

Sl. no.	RT (min)	Area	PA (%)	Compounds	MW (amu)
1	7.081	579298	0.105693	Methylcyclohexane	98.11
2	9.965	3399237	0.620188	Phenol	94.042
3	10.079	1001603	0.182742	Sulcatone	126.104
4	10.148	780660	0.142431	Butanoic acid, 2,3-dimethyl-, ethyl ester	144.115
5	10.348	865180	0.157852	1,2-Cyclohexanedione	112.052
6	11.601	1689097	0.308175	4-Methyl-1,5-heptadiene	110.11
7	16.104	925883	0.168927	Catechol	110.037
8	16.373	5726885	1.044866	Coumaran	120.058
9	18.359	4206254	0.767428	Hydroquinone	110.037
10	18.879	2153554	0.392914	p-Vinylguaiacol	150.068
11	19.412	630941	0.115115	Gamma-pyronene	136.125
12	20.916	2138840	0.39023	4-Ethylresorcinol	138.068
13	22.438	28577337	5.213916	2-(2-Hydroxy-2-phenylethyl)-3,5,6-trimethylpyrazine	242.142
14	25.225	2487140	0.453777	Ethanone, 1-(3,4-dimethoxyphenyl)-	180.079
15	25.563	4005631	0.730825	Spathulenol	220.183
16	25.694	2465579	0.449843	Caryophyllene oxide	220.183
17	26.295	639699	0.116713	Cyclopentanecarboxaldehyde, 2-methyl-3-methylene-	124.089
18	27.314	1610820	0.293893	Epiglobulol	222.198
19	27.668	1426632	0.260288	4(1H)-Pyrimidinone, 6-hydroxy-	112.027
20	28.126	1109370	0.202404	Cyclododecanone, 2-methylene-	194.167
21	28.481	1911669	0.348783	2,3-Dehydro-4-oxo-7,8-dihydro-beta-ionone	206.131
22	28.767	1292504	0.235816	Diepicedrene-1-oxide	220.183
23	29.213	10432471	1.903397	Coniferol	180.079
24	33.848	7982629	1.456425	n-Hexadecanoic acid	256.24
25	36.206	6006919	1.095958	Methyl 6,9,12-hexadecatrienoate	264.209
26	36.44	14533536	2.651634	Phytol	296.308
27	45.59	1109241	0.20238	1,3,6,10-Cyclotetradecatetraene, 3,7,11-trimethyl-14-(1-methylethyl)-, [S-(E,Z,E,E)]-	272.25
28	46.574	5222376	0.952819	(Z,E)-Farnesol	222.198
29	47.054	5942094	1.084131	Geranyl acetate	332.272
30	47.215	4894168	0.892938	Farnesol acetate	264.209
31	47.426	5740162	1.047289	3-Furaldehyde	96.021
32	47.781	1179960	0.215283	trans-13-Docosenamide	337.334
33	48.542	6365219	1.16133	Squalene	410.391
34	48.782	5112411	0.932756	2′H-Androsta-2,4,6-trieno [3,2-c]pyrazol-17.beta.-ol, 17-methyl-, acetate (ester)	366.231
35	49.686	6003161	1.095273	Spiro[2H-indole-2,8′(7′H)-[3, 7]methano[2H]furo[4,3,2-hi]indolizine]-2′a(3′H)-carboxylic acid, 4′-ethylidene-1,3,4′,5′,8′,8′b-hexahydro-3-oxo-, methyl ester	366.158
36	49.83	2892843	0.527797	Chola-5,22-dien-3-ol, (3.beta.,22Z)-	342.292
37	50.236 and 52.536	2523311 and 4290679	0.460376 and 0.782831	Curan-17-oic acid, 2,16-didehydro-20-hydroxy-19-oxo-, methyl ester	354.158
38	50.47	7587558	1.384345	Geranylgeraniol	290.261
39	50.688	12575157	2.294329	2-Hydrazino-8-hydroxy-4-phenylquinoline	251.106
40	51.477	100609821	18.35619	Hexadeca-2,6,10,14-tetraen-1-ol, 3,7,11,16-tetramethyl-, (E,E,E)-	290.261
41	52.811	4449457	0.8118	Cycloartenol	426.386
42	55.077	37780051	6.892945	Ursa-9(11),12-dien-3-ol	424.371
43	57.274	16193192	2.954437	Urs-12-ene	410.391
44	58.161	113880865	20.77749	Stigmast-4-en-3-one	412.371
45	58.55	56736847	10.3516	Ursa-9(11),12-dien-3-one	422.355
46	58.962	3509209	0.640253	C(14a)-homo-27-nor-14-beta-gammaceran-3-alpha-ol	428.402
47	59.511	11017163	2.010074	Friedelin	426.386
48	60.575	21170007	3.862453	Longipinane, (E)-	206.203
49	61.245	2733077	0.498648	Lanosterol	426.386

*Note.* MW: molecular weight; RT: retention time.

**Table 2:** KEGG analysis of the genes targeted by compounds.

Pathway ID	Pathway description	Observed gene count	False discovery rate	Matching proteins in network (labels)
05204	Chemical carcinogenesis	12	3.6*E* − 15	*SULT1A1, SULT1A2, SULT1A3, UGT1A1, UGT1A10, UGT1A3, UGT1A4, UGT1A6, UGT1A7, UGT1A8, UGT1A9, UGT2B15*
00053	Ascorbate and aldarate metabolism	9	1.21*E* − 14	*UGT1A1, UGT1A10, UGT1A3, UGT1A4, UGT1A6, UGT1A7, UGT1A8, UGT1A9, UGT2B15*
00040	Pentose and glucuronate interconversions	9	1.49*E* − 13	*UGT1A1, UGT1A10, UGT1A3, UGT1A4, UGT1A6, UGT1A7, UGT1A8, UGT1A9, UGT2B15*
00860	Porphyrin and chlorophyll metabolism	9	9.94*E* − 13	*UGT1A1, UGT1A10, UGT1A3, UGT1A4, UGT1A6, UGT1A7, UGT1A8, UGT1A9, UGT2B15*
00983	Drug metabolism—other enzymes	9	1.27*E* − 12	*UGT1A1, UGT1A10, UGT1A3, UGT1A4, UGT1A6, UGT1A7, UGT1A8, UGT1A9, UGT2B15*
00500	Starch and sucrose metabolism	9	4.62*E* − 12	*UGT1A1, UGT1A10, UGT1A3, UGT1A4, UGT1A6, UGT1A7, UGT1A8, UGT1A9, UGT2B15*
00140	Steroid hormone biosynthesis	9	6.95*E* − 12	*UGT1A1, UGT1A10, UGT1A3, UGT1A4, UGT1A6, UGT1A7, UGT1A8, UGT1A9, UGT2B15*
00830	Retinol metabolism	9	1.44*E* − 11	*UGT1A1, UGT1A10, UGT1A3, UGT1A4, UGT1A6, UGT1A7, UGT1A8, UGT1A9, UGT2B15*
00982	Drug metabolism—cytochrome P450	9	2.81*E* − 11	*UGT1A1, UGT1A10, UGT1A3, UGT1A4, UGT1A6, UGT1A7, UGT1A8, UGT1A9, UGT2B15*
00980	Metabolism of xenobiotics by cytochrome P450	9	4.52*E* − 11	*UGT1A1, UGT1A10, UGT1A3, UGT1A4, UGT1A6, UGT1A7, UGT1A8, UGT1A9, UGT2B15*
04620	Toll-like receptor signaling pathway	9	1.83*E* − 09	*CD289, IRAK4, MYD88, TIRAP, TLR3, TLR4, TLR5, TLR6, TRAF6*
05152	Tuberculosis	8	3.14*E* − 06	*CD289, IRAK2, IRAK4, MYD88, TIRAP, TLR4, TLR6, TRAF6*
**04064**	**NF-kappa B signaling pathway**	**6**	**1.35*E* − 05**	**IL1R1, IRAK4, MYD88, TIRAP, TLR4, TRAF6**
05142	Chagas disease (American trypanosomiasis)	6	2.09*E* − 05	*CD289, IRAK4, MYD88, TLR4, TLR6, TRAF6*
05133	Pertussis	5	7.9*E* − 05	*IRAK4, MYD88, TIRAP, TLR4, TRAF6*
05162	Measles	5	0.00154	*CD289, IRAK4, MYD88, TLR4, TRAF6*
05140	Leishmaniasis	4	0.00164	*IRAK4, MYD88, TLR4, TRAF6*
04210	Apoptosis	4	0.00332	*IL1R1, IRAK2, IRAK4, MYD88*
05144	Malaria	3	0.00825	*CD289, MYD88, TLR4*
05145	Toxoplasmosis	4	0.00895	*IRAK4, MYD88, TLR4, TRAF6*
05134	Legionellosis	3	0.0113	*MYD88, TLR4, TLR5*
05161	Hepatitis B	4	0.0182	*MYD88, TIRAP, TLR3, TLR4*
05164	Influenza A	4	0.0332	*IRAK4, MYD88, TLR3, TLR4*
05132	Salmonella infection	3	0.0337	*MYD88, TLR4, TLR5*
05168	Herpes simplex infection	4	0.0362	*CD289, MYD88, TLR3, TRAF6*

Bold indicates the main pathway and proteins responsible for this study.

**Table 3:** Docking scores and ligand efficiencies of compounds from the methanolic extract of *Argyreia capitiformis* stems binding with IL1R1 (PDB: 1ITB).

Compounds	IL1R1 (1ITB)			
	DS^*∗*^	MM-GBSA^*∗*^	NHA	LE^*∗*^	
Methylcyclohexane	−3.416	−8.53477	7	1.22	
Phenol	−4.567	−20.6083	7	2.94	
Sulcatone	−3.319	−22.5701	9	2.51	
Butanoic acid, 2,3-dimethyl-, ethyl ester	−3.695	−22.6538	10	2.27	
1,2-Cyclohexanedione	−3.772	−15.6443	8	1.96	
4-Methyl-1,5-heptadiene	−0.264	−15.0204	8	1.88	
Catechol	−4.755	−24.1451	8	3.02	
Coumaran	−4.368	−18.8456	9	2.09	
Hydroquinone	−4.703	−21.6152	8	2.70	
p-Vinylguaiacol	−3.965	−17.0656	11	1.55	
Gamma-pyronene	—	—	—	—	
4-Ethylresorcinol	−4.791	−19.4157	10	1.94	
2-(2-Hydroxy-2-phenylethyl)-3,5,6-trimethylpyrazine	−3.768	−18.6465	18	1.04	
Ethanone, 1-(3,4-dimethoxyphenyl)-	−5.147	−24.0973	13	1.85	
Spathulenol	—	—	—	—	
Caryophyllene oxide	—	—	—	—	
Cyclopentanecarboxaldehyde, 2-methyl-3-methylene-	−3.819	−14.8427	9	1.65	
Epiglobulol	—	—	—	—	
4(1H)-Pyrimidinone, 6-hydroxy-	−5.289	−22.2826	8	2.79	
Cyclododecanone, 2-methylene-	−3.031	−15.4537	14	1.10	
2,3-Dehydro-4-oxo-7,8-dihydro-beta-ionone	—	—	—	—	
Diepicedrene-1-oxide	−3.253	−20.532	16	1.28	
Coniferol	−4.551	−25.8365	13	1.99	
Methyl 6,9,12-hexadecatrienoate	−0.024	−27.9609	19	1.47	
Phytol	−0.765	−31.806	21	1.51	
1,3,6,10-Cyclotetradecatetraene, 3,7,11-trimethyl-14-(1-methylethyl)-, [S-(E,Z,E,E)]-	—	—	—	—	
(Z,E)-Farnesol	−1.134	−28.241	16	1.77	
Geranyl acetate	−3.607	−33.7371	24	1.41	
Farnesol, acetate	−1.636	−37.7206	24	1.57	
3-Furaldehyde	−4.663	−17.5816	7	2.51	
trans-13-Docosenamide	−3.73	−41.2676	24	1.72	
Squalene	−3.241	−26.5894	30	0.89	
Chola-5,22-dien-3-ol, (3.beta.,22Z)-	−3.62	−23.7437	25	0.95	
Curan-17-oic acid, 2,16-didehydro-20-hydroxy-19-oxo-, methyl ester	—	—	—	—	
Geranylgeraniol	−1.699	−26.6476	21	1.27	
2-Hydrazino-8-hydroxy-4-phenylquinoline	−5.348	−26.1207	19	1.37	
Cycloartenol	−2.877	−17.9477	31	0.58	
Ursa-9(11),12-dien-3-ol	—	—	—	—	
Urs-12-ene	−2.712	−25.2104	30	0.84	
Stigmast-4-en-3-one	−2.78	−23.2611	30	0.78	
Ursa-9(11),12-dien-3-one	—	—	—	—	
C(14a)-Homo-27-nor-14-beta-gammaceran-3-alpha-ol	—	—	—	—	
Friedelin	—	—	—	—	
Longipinane, (E)-	—	—	—	—	
Lanosterol	−3.249	−26.5783	31	0.86	
Aspirin	−4.26	−21.1433	13	1.63	

*Note.* DS: docking score; NHA: number of heavy atoms; LE: ligand efficiency. ^∗^Results presented in kcal/mol.

**Table 4:** Docking scores and ligand efficiencies of compounds from the methanolic extract of *Argyreia capitiformis* stems binding with IRAK4 (PDB: 6EGA).

Compounds	IRAK4 (6EGA)			
	DS^*∗*^	MM-GBSA^*∗*^	NHA	LE^*∗*^
Methylcyclohexane	−5.343	−28.5422	7	4.08
Phenol	−6.799	−30.4637	7	4.35
Sulcatone	−4.611	−34.0534	9	3.78
Butanoic acid, 2,3-dimethyl-, ethyl ester	−5.338	−27.5643	10	2.76
1,2-Cyclohexanedione	−5.827	−26.2939	8	3.29
4-Methyl-1,5-heptadiene	−2.392	−31.5398	8	3.94
Catechol	−6.363	−35.5712	8	4.45
Coumaran	−7.179	−34.9848	9	3.89
Hydroquinone	−6.568	−30.1474	8	3.77
p-Vinylguaiacol	−6.906	−45.639	11	4.15
Gamma-pyronene	−5.242	−25.8647	10	2.59
4-Ethylresorcinol	−6.961	−33.0487	10	3.30
2-(2-Hydroxy-2-phenylethyl)-3,5,6-trimethylpyrazine	−7.589	−47.6142	18	2.65
Ethanone, 1-(3,4-dimethoxyphenyl)-	−7.704	−38.3452	13	2.95
Spathulenol	—	—	—	—
Caryophyllene oxide	—	—	—	—
Cyclopentanecarboxaldehyde, 2-methyl-3-methylene-	−6.889	−34.1045	9	3.79
Epiglobulol	—	—	—	—
4(1H)-Pyrimidinone, 6-hydroxy-	−6.247	−19.6834	8	2.46
Cyclododecanone, 2-methylene-	—	—	—	—
2,3-Dehydro-4-oxo-7,8-dihydro-beta-ionone	−6.224	−26.8822	15	1.79
Diepicedrene-1-oxide	—	—	—	—
Coniferol	−7.225	−49.0664	13	3.77
Methyl 6,9,12-hexadecatrienoate	−3.841	−51.8616	19	2.73
Phytol	−4.092	−37.6416	21	1.79
1,3,6,10-Cyclotetradecatetraene, 3,7,11-trimethyl-14-(1-methylethyl)-, [S-(E,Z,E,E)]-	—	—	—	—
(Z,E)-Farnesol	−4.144	−53.635	16	3.35
Geranyl acetate	−7.613	−46.7163	24	1.95
Farnesol acetate	−4.638	−48.5373	24	2.02
3-Furaldehyde	−6.301	−28.4227	7	4.06
trans-13-Docosenamide	−6.62	−55.939	24	2.33
Squalene	−7.603	−73.1988	30	2.44
Chola-5,22-dien-3-ol, (3.beta.,22Z)-	−7.25	−36.8492	25	1.47
Curan-17-oic acid, 2,16-didehydro-20-hydroxy-19-oxo-, methyl ester	—	—	—	—
Geranylgeraniol	−4.383	−54.1471	21	2.58
2-Hydrazino-8-hydroxy-4-phenylquinoline	−6.639	−35.0611	19	1.85
Cycloartenol	−3.914	−28.0451	31	0.90
Ursa-9(11),12-dien-3-ol	—	—	—	—
Urs-12-ene	−5.048	−24.8357	30	0.83
Stigmast-4-en-3-one	−7.199	−25.6365	30	0.85
Ursa-9(11),12-dien-3-one	—	—	—	—
C(14a)-homo-27-nor-14-beta-gammaceran-3-alpha-ol	—	—	—	—
Friedelin	—	—	—	—
Longipinane, (E)-	—	—	—	—
Lanosterol	−6.524	−29.9751	31	0.97
Aspirin	−6.798	−31.0373	13	2.39

*Note.* DS: docking score; NHA: number of heavy atoms; LE: ligand efficiency. ^∗^Results presented in kcal/mol.

**Table 5:** Docking scores and ligand efficiencies of compounds from the methanolic extract of *Argyreia capitiformis* stems binding with MYD88 (PDB: 4EO7).

Compounds	MYD88 (4EO7)			
	DS^*∗*^	MM-GBSA^*∗*^	NHA	LE^*∗*^
Methylcyclohexane	−2.691	−2.45925	7	0.35
Phenol	−3.654	−5.09725	7	0.73
Sulcatone	−1.726	−8.41174	9	0.93
Butanoic acid, 2,3-dimethyl-, ethyl ester	−1.597	−2.95307	10	0.29
1,2-Cyclohexanedione	−3.341	−6.76768	8	0.85
4-Methyl-1,5-heptadiene	0.155	−14.7508	8	1.84
Catechol	−3.771	−8.25542	8	1.03
Coumaran	—	—	—	—
Hydroquinone	−3.855	−12.4109	8	1.55
p-Vinylguaiacol	−3.207	−14.8801	11	1.35
Gamma-pyronene	—	—	—	—
4-Ethylresorcinol	−4.822	−13.2875	10	1.33
2-(2-Hydroxy-2-phenylethyl)-3,5,6-trimethylpyrazine	−3.247	−32.6507	18	1.81
Ethanone, 1-(3,4-dimethoxyphenyl)-	−3.317	−10.222	13	0.79
Spathulenol	—	—	—	—
Caryophyllene oxide	−2.647	−17.446	16	1.09
Cyclopentanecarboxaldehyde, 2-methyl-3-methylene-	—	—	—	—
Epiglobulol	—	—	—	—
4(1H)-pyrimidinone, 6-hydroxy-	−4.189	−6.98163	8	0.87
Cyclododecanone, 2-methylene-	—	—	—	—
2,3-Dehydro-4-oxo-7,8-dihydro-beta-ionone	—	—	—	—
Diepicedrene-1-oxide	—	—	—	—
Coniferol	−3.03	−22.5252	13	1.73
Methyl 6,9,12-hexadecatrienoate	—	—	—	—
Phytol	0.454	−26.4647	21	1.26
1,3,6,10-Cyclotetradecatetraene, 3,7,11-trimethyl-14-(1-methylethyl)-, [S-(E,Z,E,E)]-	—	—	—	—
(Z,E)-Farnesol	0.368	−26.3771	16	1.65
Geranyl acetate	−2.457	−29.1004	24	1.21
Farnesol, acetate	0.023	−20.0217	24	0.83
3-Furaldehyde	—	—	—	—
trans-13-Docosenamide	−2.916	−42.0383	24	1.75
Squalene	−2.59	−40.4505	30	1.35
Chola-5,22-dien-3-ol, (3.beta.,22Z)-	−2.236	−7.69418	25	0.31
Curan-17-oic acid, 2,16-didehydro-20-hydroxy-19-oxo-, methyl ester	—	—	—	—
Geranylgeraniol	0.094	−25.417	21	1.21
2-Hydrazino-8-hydroxy-4-phenylquinoline	−3.068	−7.45669	19	0.39
Cycloartenol	−1.726	−21.1763	31	0.68
Ursa-9(11),12-dien-3-ol	—	—	—	—
Urs-12-ene	−1.704	−11.5462	30	0.38
Stigmast-4-en-3-one	−1.772	−19.7209	30	0.66
Ursa-9(11),12-dien-3-one	—	—	—	—
C(14a)-homo-27-nor-14-beta-gammaceran-3-alpha-ol	—	—	—	—
Friedelin	—	—	—	—
Longipinane, (E)-	−2.592	−11.7102	15	0.78
Lanosterol	−1.84	−21.6645	31	0.69
Aspirin	−1.659	−5.81427	13	0.45

*Note.* DS: docking score; NHA: number of heavy atoms; LE: ligand efficiency. ^∗^Results presented in (kcal/mol).

**Table 6:** Docking scores and ligand efficiencies of compounds from the methanolic extract of *Argyreia capitiformis* stems binding with TIRAP (PDB: 4FZ5).

Compounds	TIRAP (4FZ5)			
	DS^*∗*^	MM-GBSA^*∗*^	NHA	LE^*∗*^
Methylcyclohexane	−3.814	−19.0091	7	2.72
Phenol	−4.448	−21.4312	7	3.06
Sulcatone	−2.879	−15.5041	9	1.72
Butanoic acid, 2,3-dimethyl-, ethyl ester	−3.225	−19.8212	10	1.98
1,2-Cyclohexanedione	−4.658	−18.7199	8	2.34
4-Methyl-1,5-heptadiene	−0.875	−18.4864	8	2.31
Catechol	−4.501	−22.731	8	2.84
Coumaran	−4.133	−19.5103	9	2.17
Hydroquinone	−5.072	−21.6796	8	2.71
p-Vinylguaiacol	−3.458	−17.6974	11	1.61
Gamma-pyronene	−3.944	−18.1058	10	1.81
4-Ethylresorcinol	−5.971	−29.5938	10	2.96
2-(2-Hydroxy-2-phenylethyl)-3,5,6-trimethylpyrazine	−4.304	−25.9223	18	1.44
Ethanone, 1-(3,4-dimethoxyphenyl)-	−5.243	−24.616	13	1.89
Spathulenol	—	—	—	—
Caryophyllene oxide	—	—	—	—
Cyclopentanecarboxaldehyde, 2-methyl-3-methylene-	−4.868	−20.8151	9	2.31
Epiglobulol	—	—	—	—
4(1H)-pyrimidinone, 6-hydroxy-	−4.845	−15.1979	8	1.89
Cyclododecanone, 2-methylene-	−2.974	−20.1319	14	1.44
2,3-Dehydro-4-oxo-7,8-dihydro-beta-ionone	−4.032	−21.7031	15	1.45
Diepicedrene-1-oxide	—	—	—	—
Coniferol	−4.855	−32.2717	13	2.48
Methyl 6,9,12-hexadecatrienoate	0.105	−31.8802	19	1.68
Phytol	0.856	−29.859	21	1.42
1,3,6,10-Cyclotetradecatetraene, 3,7,11-trimethyl-14-(1-methylethyl)-, [S-(E,Z,E,E)]-	—	—	—	—
(Z,E)-Farnesol	0.289	−15.1735	16	0.95
Geranyl acetate	−1.574	−25.783	24	1.07
Farnesol, acetate	0.333	−14.545	24	0.61
3-Furaldehyde	−4.332	−17.5272	7	2.50
trans-13-Docosenamide	−1.763	−31.4792	24	1.31
Squalene	−1.678	−23.5817	30	0.79
Chola-5,22-dien-3-ol, (3.beta.,22Z)-	−2.807	−23.4983	25	0.94
Curan-17-oic acid, 2,16-didehydro-20-hydroxy-19-oxo-, methyl ester	—	—	—	—
Geranylgeraniol	0.085	−9.16848	21	0.44
2-Hydrazino-8-hydroxy-4-phenylquinoline	−4.084	−24.5226	19	1.29
Cycloartenol	−1.963	−29.9984	31	0.97
Ursa-9(11),12-dien-3-ol	—	—	—	—
Urs-12-ene	−2.561	−20.6408	30	0.69
Stigmast-4-en-3-one	—	—	—	—
Ursa-9(11),12-dien-3-one	−3.348	−39.9359	31	1.29
C(14a)-homo-27-nor-14-beta-gammaceran-3-alpha-ol	—	—	—	—
Friedelin	—	—	—	—
Longipinane, (E)-	—	—	—	—
Lanosterol	−1.716	−27.5587	31	0.89
Aspirin	−4.343	−23.6086	13	1.82

*Note.* DS: docking score; NHA: number of heavy atoms; LE: ligand efficiency. ^*∗*^Results presented in kcal/mol.

**Table 7:** Docking scores and ligand efficiencies of compounds from the methanolic extract of *Argyreia capitiformis* stems binding with TLR4 (PDB: 3FXI).

Compounds	TLR4 (3FXI)			
	DS^*∗*^	MM-GBSA^*∗*^	NHA	LE^*∗*^
Methylcyclohexane	−5.225	−25.2088	7	3.60
Phenol	−6.387	−32.1845	7	4.59
Sulcatone	−5.227	−37.5653	9	4.17
Butanoic acid, 2,3-dimethyl-, ethyl ester	−4.588	−32.241	10	3.22
1,2-Cyclohexanedione	−6.231	−26.8612	8	3.36
4-Methyl-1,5-heptadiene	−2.573	−35.6785	8	4.45
Catechol	−6.224	−35.1395	8	4.39
Coumaran	−6.217	−31.1228	9	3.46
Hydroquinone	−5.879	−32.0765	8	4.01
p-Vinylguaiacol	−6.497	−41.7567	11	3.79
Gamma-pyronene	−5.859	−28.5764	10	2.86
4-Ethylresorcinol	−7.636	−30.7191	10	3.07
2-(2-Hydroxy-2-phenylethyl)-3,5,6-trimethylpyrazine	−7.775	−47.2615	18	2.63
Ethanone, 1-(3,4-dimethoxyphenyl)-	−7.127	−35.528	13	2.73
Spathulenol	—	—	—	—
Caryophyllene oxide	—	—	—	—
Cyclopentanecarboxaldehyde, 2-methyl-3-methylene-	−6.018	−29.885	9	3.32
Epiglobulol	—	—	—	—
4(1H)-pyrimidinone, 6-hydroxy-	−5.805	−25.5615	8	3.19
Cyclododecanone, 2-methylene-	—	—	—	—
2,3-Dehydro-4-oxo-7,8-dihydro-.beta.-ionone	−6.232	−10.5536	15	0.70
Diepicedrene-1-oxide	—	—	—	—
Coniferol	−7.058	−52.0494	13	4.003
Methyl 6,9,12-hexadecatrienoate	−2.949	−48.4222	19	2.55
Phytol	−3.527	−48.9579	21	2.33
1,3,6,10-Cyclotetradecatetraene, 3,7,11-trimethyl-14-(1-methylethyl)-, [S-(E,Z,E,E)]-	—	—	—	—
(Z,E)-Farnesol	−3.612	−49.9903	16	3.12
Geranyl acetate	−6.867	−47.3758	24	1.97
Farnesol, acetate	−4.062	−56.2792	24	2.34
3-Furaldehyde	−5.191	−27.4652	7	3.92
trans-13-Docosenamide	−5.443	−52.2716	24	2.18
Squalene	−6.729	−40.8575	30	1.36
Chola-5,22-dien-3-ol, (3.beta.,22Z)-	—	—	—	—
Curan-17-oic acid, 2,16-didehydro-20-hydroxy-19-oxo-, methyl ester	—	—	—	—
Geranylgeraniol	−3.692	−42.9074	21	2.04
2-Hydrazino-8-hydroxy-4-phenylquinoline	−5.647	0.186151	19	−0.009
Cycloartenol	—	—	—	—
Ursa-9(11),12-dien-3-ol	—	—	—	—
Urs-12-ene	—	—	—	—
Stigmast-4-en-3-one	—	—	—	—
Ursa-9(11),12-dien-3-one	—	—	—	—
C(14a)-homo-27-nor-14-beta-gammaceran-3-alpha-ol	—	—	—	—
Friedelin	—	—	—	—
Longipinane, (E)-	—	—	—	—
Lanosterol	—	—	—	—
Aspirin	−6.329	−35.694	13	2.75

*Note.* DS: docking score; NHA: number of heavy atoms; LE: ligand efficiency. ^∗^Results presented in kcal/mol.

**Table 8:** Docking scores and ligand efficiencies of compounds from the methanolic extract of *Argyreia capitiformis* stems binding with TRAF6 (PDB: 3HCT).

Compounds	TRAF6 (3HCT)			
	DS^*∗*^	MM-GBSA^*∗*^	NHA	LE^*∗*^
Methylcyclohexane	−4.23	−15.5462	7	2.22
Phenol	−4.75	−17.0982	7	2.44
Sulcatone	−3.224	−22.1931	9	2.47
Butanoic acid, 2,3-dimethyl-, ethyl ester	−3.328	−17.6969	10	1.77
1,2-Cyclohexanedione	−5.406	−17.7531	8	2.22
4-Methyl-1,5-heptadiene	−1.225	−18.4899	8	2.31
Catechol	−4.53	−18.205	8	2.28
Coumaran	−3.768	−20.8505	9	2.32
Hydroquinone	−4.795	−18.3408	8	2.29
p-Vinylguaiacol	−4.303	−17.778	11	1.62
Gamma-pyronene	−4.133	−21.4044	10	2.14
4-Ethylresorcinol	−5.48	−19.6712	10	1.97
2-(2-Hydroxy-2-phenylethyl)-3,5,6-trimethylpyrazine	−5.119	−41.8887	18	2.33
Ethanone, 1-(3,4-dimethoxyphenyl)-	−5.708	−29.3694	13	2.26
Spathulenol	—	—	—	—
Caryophyllene oxide	—	—	—	—
Cyclopentanecarboxaldehyde, 2-methyl-3-methylene-	−5.198	−20.7855	9	2.31
Epiglobulol	—	—	—	—
4(1H)-pyrimidinone, 6-hydroxy-	−5.483	−15.5853	8	1.95
Cyclododecanone, 2-methylene-	−4.619	−3.58279	14	0.26
2,3-Dehydro-4-oxo-7,8-dihydro-beta-ionone	−4.327	−25.9777	15	1.73
Diepicedrene-1-oxide	—	—	—	—
Coniferol	−3.698	−31.781	13	2.44
Methyl 6,9,12-hexadecatrienoate	0.144	−27.9098	19	1.47
Phytol	1.098	−20.446	21	0.97
1,3,6,10-Cyclotetradecatetraene, 3,7,11-trimethyl-14-(1-methylethyl)-, [S-(E,Z,E,E)]-	—	—	—	—
(Z,E)-Farnesol	−0.033	−18.0454	16	1.13
Geranyl acetate	−2.742	−22.1469	24	0.92
Farnesol, acetate	−0.582	−27.6353	24	1.15
3-Furaldehyde	−4.911	−16.8696	7	2.41
trans-13-Docosenamide	−2.779	−43.4455	24	1.81
Squalene	−2.1	−40.8237	30	1.36
Chola-5,22-dien-3-ol, (3.beta.,22Z)-	−2.904	−21.7666	25	0.87
Curan-17-oic acid, 2,16-didehydro-20-hydroxy-19-oxo-, methyl ester	—	—	—	—
Geranylgeraniol	−0.313	−26.3103	21	1.253
2-Hydrazino-8-hydroxy-4-phenylquinoline	−4.282	−21.7512	19	1.14
Cycloartenol	−2.32	−17.571	31	0.57
Ursa-9(11),12-dien-3-ol	—	—	—	—
Urs-12-ene	−2.153	−13.0659	30	0.44
Stigmast-4-en-3-one	−2.229	−18.7231	30	0.62
Ursa-9(11),12-dien-3-one	—	—	—	—
C(14a)-homo-27-nor-14-beta-gammaceran-3.alpha.-ol	—	—	—	—
Friedelin	—	—	—	—
Longipinane, (E)-	—	—	—	—
Lanosterol	−2.204	−16.5677	31	0.53
Aspirin	−4.81	−26.889	13	2.07

*Note.* DS: docking score; NHA: number of heavy atoms; LE: ligand efficiency. ^∗^Results presented in kcal/mol.

## Data Availability

The data are available within the manuscript and also accessible from the corresponding author upon request.

## Abbreviations

GC-MS: Gas chromatography-mass spectrometry
KEGG: Kyoto Encyclopedia of Genes and Genomes
WHO: World Health Organization
NF-*κ*B: Nuclear factor-kappa B
STRING: Search Tool for Retrieval of Interacting Genes
PPIs: Protein-protein interactions
IL: Interleukins
PKC: Protein kinase C
MAPK: Mitogen-activated protein kinase
IRF: Interferon regulatory factor
IRAK: Receptor-associated kinase
TLR: Toll-like receptor
3D: Three-dimensional
PDB: Protein Data Bank
RMSD: Root-mean-square deviation of the complexes
SASA: Solvent accessible surface area
Rg: Radius of gyration.

## Supplementary Materials

Supplementary Materials Table S1: biological processes of the genes targeted by the compounds; Table S2: molecular functions of the genes interacting with the compounds; Table S3: protein-protein interaction (PPI) status of 10 proteins with coexpression; Table S4: molecular interactions with the best ligand efficiencies for compounds binding with IL1R1 (PDB: 1ITB); Table S5: molecular interactions with the best ligand efficiencies for compounds binding with IRAK4 (PDB: 6EGA); Table S6: molecular interactions with the best ligand efficiencies for compounds binding with MYD88 (4EO7); Table S7: molecular interactions with the best ligand efficiencies for compounds binding with TIRAP (PDB: 4FZ5); Table S8: molecular interactions with the best ligand efficiencies for compounds binding with TLR4 (PDB: 3FXI); and Table S9: molecular interactions with the best ligand efficiencies for compounds binding with TRAF6 (PDB: 3HCT). Figure S1: GC-MS chromatograph of the methanolic extract of *Argyreia capitiformis* stem; Figure S2: protein-protein interaction (PPI) network of 10 proteins; Figure S3: gene co-occurrence of 10 proteins for *Homo sapiens*; Figure S4: gene coexpression of 10 proteins in PPI network; and Figure S5: two-dimensional representations of the best ligand efficiencies for compounds binding with IL1R1 (PDB: 1ITB). 3-Furaldehyde (A), 4(1H)-pyrimidinone, 6-hydroxy- (B), catechol (C), hydroquinone (D), phenol (E), sulcatone (F), and aspirin (G) are shown. Figure S6: two-dimensional representations of the best ligand efficiencies for compounds binding to IRAK4 (PDB: 6EGA). 3-Furaldehyde (A), 4-methyl-1,5-heptadiene (B), catechol (C), methylcyclohexane (D), p-vinylguaiacol (E), phenol (F), and aspirin (G) are shown. Figure S7: two-dimensional representations of the best ligand efficiencies for compounds binding to MYD88 (4EO7). (Z,E)-farnesol (A), 2-(2-hydroxy-2-phenylethyl)-3,5,6-trimethylpyrazine (B), 4-methyl-1,5-heptadiene (C), coniferol (D), hydroquinone (E), trans-13-docosenamide (F), and aspirin (G) are shown. Figure S8: two-dimensional representations of the best ligand efficiencies for compounds binding to TIRAP (PDB: 4FZ5). 3-Furaldehyde (A), 4-ethylresorcinol (B), catechol (C), hydroquinone (D), methylcyclohexane (E), phenol (F), and aspirin (G) are shown. Figure S9: two-dimensional representations of the best ligand efficiencies for compounds against TLR4 (PDB: 3FXI). 4-Methyl-1,5-heptadiene (A), catechol (B), coniferol (C), hydroquinone (D), phenol (E), sulcatone (F), and aspirin (G) are shown. Figure S10: two-dimensional representations of the best ligand efficiencies for compounds against TRAF6 (PDB: 3HCT). 2-(2-Hydroxy-2-phenylethyl)-3,5,6-trimethylpyrazine (A), 3-furaldehyde (B), coniferol (C), coumaran (D), phenol (E), sulcatone (F), and aspirin (G) are shown.
