# Supplementary material for: Network Pharmacology- and Molecular Docking-Based Identification of Potential Phytocompounds from Argyreia capitiformis in the Treatment of Inflammation
Source: Evid Based Complement Alternat Med. 2022 Jan 31;2022:8037488. doi: 10.1155/2022/8037488 (PMC8820870; doi:10.1155/2022/8037488)
Supplement: Supplementary Materials — Table S1: biological processes of the genes targeted by the compounds; Table S2: molecular functions of the genes interacting with the compounds; Table S3: protein-protein interaction (PPI) status of 10 proteins with coexpression; Table S4: molecular interactions with the best ligand efficiencies for compounds binding with IL1R1 (PDB: 1ITB); Table S5: molecular interactions with the best ligand efficiencies for compounds binding with IRAK4 (PDB: 6EGA); Table S6: molecular interactions with the best ligand efficiencies for compounds binding with MYD88 (4EO7); Table S7: molecular interactions with the best ligand efficiencies for compounds binding with TIRAP (PDB: 4FZ5); Table S8: molecular interactions with the best ligand efficiencies for compounds binding with TLR4 (PDB: 3FXI); and Table S9: molecular interactions with the best ligand efficiencies for compounds binding with TRAF6 (PDB: 3HCT). Figure S1: GC-MS chromatograph of the methanolic extract of Argyreia capitiformis stem; Figure S2: protein-protein interaction (PPI) network of 10 proteins; Figure S3: gene co-occurrence of 10 proteins for Homo sapiens; Figure S4: gene coexpression of 10 proteins in PPI network; and Figure S5: two-dimensional representations of the best ligand efficiencies for compounds binding with IL1R1 (PDB: 1ITB). 3-Furaldehyde (A), 4(1H)-pyrimidinone, 6-hydroxy- (B), catechol (C), hydroquinone (D), phenol (E), sulcatone (F), and aspirin (G) are shown. Figure S6: two-dimensional representations of the best ligand efficiencies for compounds binding to IRAK4 (PDB: 6EGA). 3-Furaldehyde (A), 4-methyl-1,5-heptadiene (B), catechol (C), methylcyclohexane (D), p-vinylguaiacol (E), phenol (F), and aspirin (G) are shown. Figure S7: two-dimensional representations of the best ligand efficiencies for compounds binding to MYD88 (4EO7). (Z,E)-farnesol (A), 2-(2-hydroxy-2-phenylethyl)-3,5,6-trimethylpyrazine (B), 4-methyl-1,5-heptadiene (C), coniferol (D), hydroquinone (E), trans-13-docosenamide (F), and [file 8037488.f1.docx]

**Supplementary Materials**

Table S1. Biological processes of the genes targeted by the compounds.

| **Pathway ID** | **Pathway Description** | **Observed Gene Count** | **False Discovery Rate** | **Matching Proteins in Network (Labels)** |
| --- | --- | --- | --- | --- |
| GO.0009812 | flavonoid metabolic process | 13 | 2.97E-21 | *SULT1A1,SULT1A3,SULT1A4,SULT1B1,UGT1A1,UGT1A10,*  *UGT1A3,UGT1A4,UGT1A6,UGT1A7,UGT1A8,UGT1A9,UGT2B15* |
| GO.1904224 | negative regulation of glucuronosyltransferase activity | 8 | 4.83E-17 | *UGT1A1,UGT1A10,UGT1A3,UGT1A4,*  *UGT1A6,UGT1A7,UGT1A8,UGT1A9* |
| GO.2001030 | negative regulation of cellular glucuronidation | 8 | 4.83E-17 | *UGT1A1,UGT1A10,UGT1A3,UGT1A4,*  *UGT1A6,UGT1A7,UGT1A8,UGT1A9* |
| GO.0052697 | xenobiotic glucuronidation | 8 | 2.89E-16 | *UGT1A1,UGT1A10,UGT1A3,UGT1A4,*  *UGT1A6,UGT1A7,UGT1A8,UGT1A9* |
| GO.0006805 | xenobiotic metabolic process | 15 | 1.98E-14 | *CYP51A1,SULT1A1,SULT1A2,SULT1A3,SULT1A4,SULT1B1,UGT1A1,*  *UGT1A10,UGT1A3,UGT1A4,UGT1A6,*  *UGT1A7,UGT1A8,UGT1A9,UGT2B15* |
| GO.0009813 | flavonoid biosynthetic process | 9 | 1.98E-14 | *UGT1A1,UGT1A10,UGT1A3,UGT1A4,UGT1A6,*  *UGT1A7,UGT1A8,UGT1A9,UGT2B15* |
| GO.0052696 | flavonoid glucuronidation | 9 | 1.98E-14 | *UGT1A1,UGT1A10,UGT1A3,UGT1A4,UGT1A6,*  *UGT1A7,UGT1A8,UGT1A9,UGT2B15* |
| GO.0071466 | cellular response to xenobiotic stimulus | 15 | 2.56E-14 | *CYP51A1,SULT1A1,SULT1A2,SULT1A3,SULT1A4,SULT1B1,UGT1A1,UGT1A10,*  *UGT1A3,UGT1A4,UGT1A6,UGT1A7,UGT1A8,UGT1A9,UGT2B15* |
| GO.0045922 | negative regulation of fatty acid metabolic process | 8 | 1.91E-11 | *UGT1A1,UGT1A10,UGT1A3,UGT1A4,*  *UGT1A6,UGT1A7,UGT1A8,UGT1A9* |
| GO.0051552 | flavone metabolic process | 5 | 2.12E-09 | *UGT1A1,UGT1A10,UGT1A7,UGT1A8,UGT1A9* |
| GO.0019217 | regulation of fatty acid metabolic process | 9 | 7.52E-09 | *CPT1A,UGT1A1,UGT1A10,UGT1A3,UGT1A4,*  *UGT1A6,UGT1A7,UGT1A8,UGT1A9* |
| **GO.0002755** | **MyD88-dependent toll-like receptor signaling pathway** | **8** | **2.19E-07** | ***IRAK2,IRAK4,MYD88,TIRAP,TLR4,TLR5,TLR6,TRAF6*** |
| **GO.0034142** | **toll-like receptor 4 signaling pathway** | **8** | **9.14E-07** | ***IRAK2,IRAK4,MYD88,TIRAP,TLR3,TLR4,TLR6,TRAF6*** |
| GO.0051923 | sulfation | 5 | 1.11E-06 | *SULT1A1,SULT1A2,SULT1A3,SULT1A4,SULT1B1* |
| GO.0042573 | retinoic acid metabolic process | 5 | 1.5E-06 | *UGT1A1,UGT1A3,UGT1A7,UGT1A8,UGT1A9* |
| GO.0005996 | monosaccharide metabolic process | 10 | 1.7E-06 | *CPT1A,UGT1A1,UGT1A10,UGT1A3,UGT1A4,UGT1A6,*  *UGT1A7,UGT1A8,UGT1A9,UGT2B15* |
| **GO.0038123** | **toll-like receptor TLR1:TLR2 signaling pathway** | **7** | **2.31E-06** | ***IRAK2,IRAK4,MYD88,TIRAP,TLR4,TLR6,TRAF6*** |
| **GO.0038124** | **toll-like receptor TLR6:TLR2 signaling pathway** | **7** | **2.31E-06** | ***IRAK2,IRAK4,MYD88,TIRAP,TLR4,TLR6,TRAF6*** |
| GO.0050427 | 3 -phosphoadenosine 5 -phosphosulfate metabolic process | 5 | 2.32E-06 | *SULT1A1,SULT1A2,SULT1A3,SULT1A4,SULT1B1* |
| **GO.0034134** | **toll-like receptor 2 signaling pathway** | **7** | **2.54E-06** | ***IRAK2,IRAK4,MYD88,TIRAP,TLR4,TLR6,TRAF6*** |
| **GO.0002224** | **toll-like receptor signaling pathway** | **8** | **3.15E-06** | ***IRAK2,IRAK4,MYD88,TIRAP,TLR3,TLR4,TLR5,TRAF6*** |
| GO.0042440 | pigment metabolic process | 6 | 1.43E-05 | *UGT1A1,UGT1A10,UGT1A4,UGT1A7,UGT1A8,UGT1A9* |
| **GO.0032755** | **positive regulation of interleukin-6 production** | **6** | **2.06E-05** | ***MYD88,TIRAP,TLR3,TLR4,TLR6,TRAF6*** |
| GO.0008202 | steroid metabolic process | 9 | 2.78E-05 | *CYP51A1,SULT1A1,SULT1A2,SULT1A3,SULT1A4,*  *SULT1B1,UGT1A1,UGT1A8,UGT2B15* |
| GO.0043436 | oxoacid metabolic process | 15 | 5.73E-05 | *CPT1A,SULT1A1,SULT1A2,SULT1A3,SULT1A4,SULT1B1,UGT1A1,UGT1A10,*  *UGT1A3,UGT1A4,UGT1A6,UGT1A7,UGT1A8,UGT1A9,UGT2B15* |
| GO.0070887 | cellular response to chemical stimulus | 23 | 8.18E-05 | *CPT1A,CYP51A1,IL1R1,IRAK2,IRAK4,MYD88,SULT1A1,SULT1A2,SULT1A3,SULT1A4,SULT1B1,TIRAP,TLR4,*  *TLR5,UGT1A1,UGT1A10,UGT1A3,UGT1A4,UGT1A6,UGT1A7,UGT1A8,UGT1A9,UGT2B15* |
| GO.0034754 | cellular hormone metabolic process | 6 | 0.000129 | *SULT1A1,UGT1A1,UGT1A3,UGT1A7,UGT1A8,UGT1A9* |
| GO.0042445 | hormone metabolic process | 7 | 0.000217 | *SULT1A1,SULT1B1,UGT1A1,UGT1A3,UGT1A7,UGT1A8,UGT1A9* |
| GO.0045410 | positive regulation of interleukin-6 biosynthetic process | 3 | 0.000383 | *TIRAP,TLR6,TRAF6* |
| **GO.0007249** | **I-kappaB kinase/NF-kappaB signaling** | **5** | **0.000393** | ***IRAK2,TIRAP,TLR3,TLR4,TRAF6*** |
| **GO.0043123** | **positive regulation of I-kappaB kinase/NF-kappaB signaling** | **7** | **0.000601** | ***IRAK4,MYD88,TIRAP,TLR3,TLR4,TLR6,TRAF6*** |
| **GO.0034146** | **toll-like receptor 5 signaling pathway** | **5** | **0.000712** | ***IRAK2,IRAK4,MYD88,TLR5,TRAF6*** |
| **GO.0034166** | **toll-like receptor 10 signaling pathway** | **5** | **0.000712** | ***IRAK2,IRAK4,MYD88,TLR5,TRAF6*** |
| **GO.0032735** | **positive regulation of interleukin-12 production** | **4** | **0.000718** | ***TIRAP,TLR3,TLR4,TRAF6*** |
| GO.0032787 | monocarboxylic acid metabolic process | 10 | 0.000718 | *CPT1A,UGT1A1,UGT1A10,UGT1A3,UGT1A4,*  *UGT1A6,UGT1A7,UGT1A8,UGT1A9,UGT2B15* |
| GO.0051338 | regulation of transferase activity | 13 | 0.00101 | *IRAK2,TLR3,TLR4,TLR6,TRAF6,UGT1A1,UGT1A10,UGT1A3,*  *UGT1A4,UGT1A6,UGT1A7,UGT1A8,UGT1A9* |
| GO.0018958 | phenol-containing compound metabolic process | 5 | 0.00137 | *SULT1A1,SULT1A2,SULT1A3,SULT1A4,SULT1B1* |
| GO.0071219 | cellular response to molecule of bacterial origin | 6 | 0.00137 | *IRAK2,TIRAP,TLR4,TLR5,TLR6,TRAF6* |
| **GO.0070498** | **interleukin-1-mediated signaling pathway** | **3** | **0.00186** | ***IL1R1,IRAK2,TRAF6*** |
| **GO.0032757** | **positive regulation of interleukin-8 production** | **4** | **0.00191** | ***TIRAP,TLR3,TLR4,TLR5*** |
| GO.0006584 | catecholamine metabolic process | 4 | 0.00228 | *SULT1A1,SULT1A2,SULT1A3,SULT1A4* |
| GO.0009617 | response to bacterium | 9 | 0.00327 | *IRAK2,MYD88,TIRAP,TLR3,TLR4,TLR5,TLR6,TRAF6,UGT1A1* |
| GO.0042088 | T-helper 1 type immune response | 3 | 0.00328 | *TLR4,TLR6,TRAF6* |
| **GO.0034123** | **positive regulation of toll-like receptor signaling pathway** | **3** | **0.00536** | ***TIRAP,TLR3,TLR5*** |
| **GO.0046330** | **positive regulation of JNK cascade** | **5** | **0.00586** | ***TIRAP,TLR3,TLR4,TLR6,TRAF6*** |
| **GO.0007250** | **activation of NF-kappaB-inducing kinase activity** | **3** | **0.00605** | ***TLR3,TLR6,TRAF6*** |
| GO.0002237 | response to molecule of bacterial origin | 7 | 0.0071 | *IRAK2,TIRAP,TLR4,TLR5,TLR6,TRAF6,UGT1A1* |
| GO.0042742 | defense response to bacterium | 6 | 0.0071 | *MYD88,TIRAP,TLR3,TLR4,TLR5,TLR6* |
| GO.0010033 | response to organic substance | 20 | 0.00749 | *CPT1A,IL1R1,IRAK2,IRAK4,MYD88,SULT1A3,SULT1A4,TIRAP,TLR3,TLR4,TLR5,TLR6,*  *UGT1A10,UGT1A3,UGT1A4,UGT1A6,UGT1A7,UGT1A8,UGT1A9,UGT2B15* |
| GO.0071310 | cellular response to organic substance | 17 | 0.00749 | *CPT1A,IL1R1,IRAK2,IRAK4,MYD88,SULT1A3,SULT1A4,TLR4,TLR5,UGT1A10,*  *UGT1A3,UGT1A4,UGT1A6,UGT1A7,UGT1A8,UGT1A9,UGT2B15* |
| **GO.0051092** | **positive regulation of NF-kappaB transcription factor activity** | **5** | **0.00973** | ***IRAK2,TIRAP,TLR3,TLR4,TRAF6*** |
| **GO.0006954** | **inflammatory response** | **8** | **0.0109** | ***IRAK2,MYD88,TIRAP,TLR3,TLR4,TLR5,TLR6,UGT1A1*** |
| GO.0042221 | response to chemical | 25 | 0.0131 | *CPT1A,CYP51A1,IL1R1,IRAK2,IRAK4,MYD88,SULT1A1,SULT1A2,SULT1A3,SULT1A4,SULT1B1,TIRAP,TLR3,*  *TLR4,TLR5,TLR6,UGT1A1,UGT1A10,UGT1A3,UGT1A4,UGT1A6,UGT1A7,UGT1A8,UGT1A9,UGT2B15* |
| **GO.0034162** | **toll-like receptor 9 signaling pathway** | **4** | **0.0153** | ***IRAK2,IRAK4,MYD88,TRAF6*** |
| GO.0071260 | cellular response to mechanical stimulus | 4 | 0.0153 | *MYD88,TLR3,TLR4,TLR5* |
| GO.0002730 | regulation of dendritic cell cytokine production | 2 | 0.0159 | *TLR3,TLR4* |
| GO.0071221 | cellular response to bacterial lipopeptide | 2 | 0.0159 | *TIRAP,TLR6* |
| GO.0006629 | lipid metabolic process | 12 | 0.0173 | *CYP51A1,SULT1A1,SULT1A2,SULT1A3,SULT1A4,SULT1B1,*  *UGT1A1,UGT1A3,UGT1A7,UGT1A8,UGT1A9,UGT2B15* |
| **GO.0038061** | **NIK/NF-kappaB signaling** | **3** | **0.0182** | ***TLR3,TLR6,TRAF6*** |
| **GO.0035666** | **TRIF-dependent toll-like receptor signaling pathway** | **4** | **0.0186** | ***IRAK2,TLR3,TLR4,TRAF6*** |
| **GO.0034138** | **toll-like receptor 3 signaling pathway** | **4** | **0.0223** | ***IRAK2,TLR3,TLR4,TRAF6*** |
| GO.0042167 | heme catabolic process | 2 | 0.0286 | *UGT1A1,UGT1A4* |
| GO.0045084 | positive regulation of interleukin-12 biosynthetic process | 2 | 0.0286 | *TLR4,TRAF6* |
| GO.0045359 | positive regulation of interferon-beta biosynthetic process | 2 | 0.0286 | *TLR3,TLR4* |
| GO.0032496 | response to lipopolysaccharide | 6 | 0.0342 | *IRAK2,TIRAP,TLR4,TLR5,TRAF6,UGT1A1* |
| GO.0009804 | coumarin metabolic process | 2 | 0.0362 | *UGT1A7,UGT1A8* |
| **GO.0032667** | **regulation of interleukin-23 production** | **2** | **0.0362** | ***MYD88,TLR4*** |
| **GO.0032648** | **regulation of interferon-beta production** | **3** | **0.0413** | ***TIRAP,TLR3,TLR4*** |
| GO.0032870 | cellular response to hormone stimulus | 8 | 0.0413 | *UGT1A10,UGT1A3,UGT1A4,UGT1A6,*  *UGT1A7,UGT1A8,UGT1A9,UGT2B15* |
| GO.0032722 | positive regulation of chemokine production | 3 | 0.0427 | *TIRAP,TLR3,TLR4* |
| GO.0071223 | cellular response to lipoteichoic acid | 2 | 0.0427 | *TIRAP,TLR4* |
| GO.0017144 | drug metabolic process | 3 | 0.0482 | *UGT1A1,UGT1A7,UGT1A8* |

**Table S2.** Molecular functions of the genes interacting with the compounds.

| **Pathway ID** | **Pathway Description** | **Gene Count** | **False Discovery Rate** | **Matching Proteins in Network (Labels)** |
| --- | --- | --- | --- | --- |
| GO.0015020 | glucuronosyltransferase activity | 9 | 8.7E-12 | *UGT1A1,UGT1A10,UGT1A3,UGT1A4,*  *UGT1A6,UGT1A7,UGT1A8,UGT1A9,UGT2B15* |
| GO.0004062 | aryl sulfotransferase activity | 5 | 5.37E-09 | *SULT1A1,SULT1A2,SULT1A3,SULT1A4,SULT1B1* |
| **GO.0001972** | **retinoic acid binding** | **5** | **2.69E-06** | ***UGT1A1,UGT1A3,UGT1A7,UGT1A8,UGT1A9*** |
| GO.0046982 | protein heterodimerization activity | 10 | 3.03E-06 | *IRAK2,TIRAP,UGT1A1,UGT1A10,UGT1A3,*  *UGT1A4,UGT1A6,UGT1A7,UGT1A8,UGT1A9* |
| GO.0042802 | identical protein binding | 13 | 0.00421 | *IRAK2,MYD88,TIRAP,TLR3,TRAF6,UGT1A1,UGT1A10,*  *UGT1A3,UGT1A4,UGT1A6,UGT1A7,UGT1A8,UGT1A9* |
| GO.0042803 | protein homodimerization activity | 10 | 0.00421 | *IRAK2,TIRAP,UGT1A1,UGT1A10,UGT1A3,*  *UGT1A4,UGT1A6,UGT1A7,UGT1A8,UGT1A9* |
| GO.0008146 | sulfotransferase activity | 4 | 0.00564 | *SULT1A1,SULT1A2,SULT1A3,SULT1A4* |
| GO.0047894 | flavonol 3-sulfotransferase activity | 2 | 0.0104 | *SULT1A1,SULT1A2* |
| **GO.0005080** | **protein kinase C binding** | **3** | **0.0447** | ***TIRAP,UGT1A10,UGT1A7*** |

Table S3. Protein–protein interaction (PPI) status of 10 proteins with co-expression.

| **Node1** | **Node2** | **Neighborhood on Chromosome** | **Gene Fusion** | **Phylogenetic Cooccurrence** | **Homology** | **Coexpression** | **Experimentally Determined Interaction** | **Database Annotated** | **Automated Textmining** | **Combined Score** |
| --- | --- | --- | --- | --- | --- | --- | --- | --- | --- | --- |
| IL1R1 | IRAK2 | 0 | 0 | 0 | 0 | 0 | 0.384 | 0.900 | 0.669 | 0.977 |
| IL1R1 | TLR3 | 0 | 0 | 0 | 0 | 0 | 0 | 0 | 0.734 | 0.734 |
| IL1R1 | TLR4 | 0 | 0 | 0 | 0 | 0.083 | 0 | 0 | 0.798 | 0.807 |
| IL1R1 | TIRAP | 0 | 0 | 0 | 0 | 0 | 0 | 0 | 0.671 | 0.671 |
| IL1R1 | TLR6 | 0 | 0 | 0 | 0 | 0 | 0 | 0 | 0.705 | 0.705 |
| IL1R1 | TLR5 | 0 | 0 | 0 | 0 | 0.061 | 0 | 0 | 0.709 | 0.715 |
| IL1R1 | TRAF6 | 0 | 0 | 0 | 0 | 0 | 0.993 | 0.900 | 0.774 | 0.999 |
| IL1R1 | MYD88 | 0 | 0 | 0 | 0 | 0.061 | 0.993 | 0.900 | 0.915 | 0.999 |
| IL1R1 | IRAK4 | 0 | 0 | 0 | 0 | 0.061 | 0.993 | 0.900 | 0.749 | 0.999 |
| IRAK2 | TLR5 | 0 | 0 | 0 | 0 | 0 | 0.177 | 0 | 0.566 | 0.627 |
| IRAK2 | TLR6 | 0 | 0 | 0 | 0 | 0.065 | 0.177 | 0 | 0.562 | 0.633 |
| IRAK2 | TLR3 | 0 | 0 | 0 | 0 | 0 | 0.467 | 0 | 0.595 | 0.775 |
| IRAK2 | TLR4 | 0 | 0 | 0 | 0 | 0.057 | 0.467 | 0.900 | 0.654 | 0.980 |
| IRAK2 | TIRAP | 0 | 0 | 0 | 0 | 0 | 0.501 | 0.900 | 0.651 | 0.981 |
| IRAK2 | TRAF6 | 0 | 0 | 0 | 0 | 0.059 | 0.472 | 0.900 | 0.783 | 0.987 |
| IRAK2 | IRAK4 | 0 | 0 | 0 | 0.691 | 0 | 0.958 | 0.900 | 0.905 | 0.996 |
| IRAK2 | MYD88 | 0 | 0 | 0 | 0 | 0 | 0.882 | 0.900 | 0.940 | 0.999 |
| IRAK4 | TLR3 | 0 | 0 | 0 | 0 | 0.062 | 0.177 | 0 | 0.771 | 0.808 |
| IRAK4 | TLR4 | 0 | 0 | 0 | 0 | 0.059 | 0.435 | 0.900 | 0.741 | 0.984 |
| IRAK4 | TIRAP | 0 | 0 | 0 | 0 | 0 | 0.807 | 0.900 | 0.764 | 0.995 |
| IRAK4 | TLR6 | 0 | 0 | 0 | 0 | 0.086 | 0.177 | 0 | 0.674 | 0.733 |
| IRAK4 | TLR5 | 0 | 0 | 0 | 0 | 0 | 0.177 | 0 | 0.675 | 0.721 |
| IRAK4 | TRAF6 | 0 | 0 | 0 | 0 | 0.059 | 0.765 | 0.900 | 0.901 | 0.997 |
| IRAK4 | MYD88 | 0 | 0 | 0 | 0 | 0.061 | 0.922 | 0.900 | 0.982 | 0.999 |
| MYD88 | TLR3 | 0 | 0 | 0 | 0 | 0.061 | 0.544 | 0.900 | 0.948 | 0.997 |
| MYD88 | TLR4 | 0 | 0 | 0 | 0.580 | 0.065 | 0.902 | 0.900 | 0.962 | 0.994 |
| MYD88 | TIRAP | 0 | 0 | 0 | 0.610 | 0 | 0.463 | 0.900 | 0.943 | 0.964 |
| MYD88 | TLR6 | 0 | 0 | 0 | 0 | 0.070 | 0.297 | 0.900 | 0.888 | 0.991 |
| MYD88 | TLR5 | 0 | 0 | 0 | 0 | 0.061 | 0.544 | 0.800 | 0.879 | 0.988 |
| MYD88 | TRAF6 | 0 | 0 | 0 | 0 | 0.061 | 0.876 | 0.900 | 0.945 | 0.999 |
| TIRAP | TLR3 | 0 | 0 | 0 | 0 | 0 | 0.064 | 0 | 0.750 | 0.756 |
| TIRAP | TLR4 | 0 | 0 | 0 | 0 | 0 | 0.448 | 0.900 | 0.882 | 0.992 |
| TIRAP | TLR5 | 0 | 0 | 0 | 0 | 0.061 | 0.064 | 0 | 0.708 | 0.720 |
| TIRAP | TLR6 | 0 | 0 | 0 | 0 | 0 | 0.064 | 0 | 0.718 | 0.725 |
| TIRAP | TRAF6 | 0 | 0 | 0 | 0 | 0 | 0.379 | 0.900 | 0.812 | 0.987 |
| TLR3 | TLR4 | 0 | 0 | 0 | 0.592 | 0.061 | 0 | 0 | 0.912 | 0.409 |
| TLR3 | TRAF6 | 0 | 0 | 0 | 0 | 0 | 0.993 | 0.900 | 0.755 | 0.999 |
| TLR4 | TLR5 | 0 | 0 | 0 | 0.594 | 0.062 | 0.379 | 0.800 | 0.894 | 0.919 |
| TLR4 | TLR6 | 0 | 0 | 0 | 0.589 | 0.133 | 0.313 | 0.900 | 0.878 | 0.958 |
| TLR4 | TRAF6 | 0 | 0 | 0 | 0 | 0.062 | 0.902 | 0.900 | 0.808 | 0.998 |
| TLR5 | TRAF6 | 0 | 0 | 0 | 0 | 0 | 0.079 | 0 | 0.712 | 0.723 |
| TLR6 | TRAF6 | 0 | 0 | 0 | 0 | 0 | 0.079 | 0.900 | 0.712 | 0.971 |

**Table S4.** Molecular interactions with the best ligand efficiencies for compounds binding with IL1R1 (PDB: 1ITB).

| **Ligands** | **Hydrogen Bond Interactions** | | **Hydrophobic Bond Interactions** | |
| --- | --- | --- | --- | --- |
|  | **Amino Acid Residue** | **Distance (Å)** | **Amino Acid Residue** | **Distance (Å)** |
| 3-Furaldehyde | LYS-93 | 4.78 | GLU-252 | 4.51 |
| 4(1H)-Pyrimidinone,_6-hydroxy- | GLU-252 | 5.29 | - | - |
|  | ASP-251 | 4.23 | - | - |
|  | LYS-93 | 4.83 | - | - |
|  | GLU-259 | 2.59 | - | - |
| Catechol | ASP-251 | 4.73 | - | - |
|  | LYS-93 | 5.23, 4.39 | - | - |
|  | GLU-259 | 4.02 | - | - |
|  | PRO-2 | 4.35 | - | - |
| Hydroquinone | ASP-251 | 4.42 | - | - |
|  | LYS-93 | 4.73 | - | - |
| Phenol | PRO-2 | 4.44 | - | - |
|  | LYS-93 | 4.44 | - | - |
| Sulcatone | LYS-93 | 4.84 | - | - |
|  | GLU-259 | 3.62 | - | - |
| Aspirin | ASP-254 | 3.76 | GLU-252 | 4.59 |
|  | LEU-257 | 6.37 | GLY-258 | 4.12, 4.74 |
|  | LYS-93 | 4.54 |  |  |

**Table S5.** Molecular interactions with the best ligand efficiencies for compounds binding with IRAK4 (PDB: 6EGA).

| **Ligands** | **Hydrogen Bond Interactions** | | **Hydrophobic Bond Interactions** | |
| --- | --- | --- | --- | --- |
|  | **Amino Acid Residue** | **Distance (Å)** | **Amino Acid Residue** | **Distance (Å)** |
| 3-Furaldehyde | MET-265 | 4.00 | VAL-263 | 5.08 |
|  | - | - | VAL-246 | 4.34 |
|  | - | - | ALA-211 | 5.34 |
|  | - | - | LEU-318 | 6.31 |
| 4-Methyl-1,5-Heptadiene | - | - | ALA-211 | 4.31, 4.68 |
|  | - | - | VAL-200 | 4.77 |
|  | - | - | TYR-262 | 4.31, 4.81, 5.01 |
|  | - | - | LEU-318 | 5.91 |
|  | - | - | MET-265 | 5.11 |
|  | - | - | VAL-246 | 5.40, 5.24 |
| Catechol | LYS-213 | 4.59 | TYR-262 | 4.91 |
|  | ASP-329 | 4.02, 3.48 | - | - |
| Methylcyclohexane | - | - | PHE-330 | 5.68, 6.22 |
|  | - | - | TYR-262 | 4.14 |
|  | - | - | LEU-318 | 5.93 |
|  | - | - | ALA-211 | 4.95 |
|  | - | - | LYS-213 | 6.63 |
| p-Vinylguaiacol | ASP-329 | 4.05, 4.06 | TYR-262 | 4.13, 4.27 |
|  | LYS-213 | 4.60 | MET-265 | 5.49 |
|  | - | - | LEU-318 | 6.11 |
|  | - | - | VAL-246 | 4.60, 5.69 |
|  | - | - | ALA-211 | 5.02 |
| Phenol | ASP-329 | 3.49 | VAL-246 | 5.55 |
|  | - | - | TYR-262 | 4.90 |
| Aspirin | ASP-329 | 4.24 | ALA-211 | 5.78 |
|  | - | - | VAL-246 | 5.20 |
|  | - | - | PHE-330 | 5.80 |
|  | - | - | TYR-262 | 3.84 |

**Table S6.** Molecular interactions with the best ligand efficiencies for compounds binding with MYD88 (4EO7).

| **Ligands** | **Hydrogen Bond Interactions** | | **Hydrophobic Bond Interactions** | |
| --- | --- | --- | --- | --- |
|  | **Amino Acid Residue** | **Distance (Å)** | **Amino Acid Residue** | **Distance (Å)** |
| Coniferol | ASP-156 | 3.06 | VAL-155 | 3.62, 3.64 |
|  | - | - | MET-157 | 4.33 |
| 2-(2-Hydroxy-2-phenylethyl)-3,5,6-trimethylpyrazine | TRP-205 | 5.45 | ARG-188 | 4.59 |
|  | ASP-156 | 3.84 | CYS-203 | 5.72 |
|  | - | - | LEU-189 | 6.03 |
|  | - | - | PRO-158 | 4.35 |
|  | - | - | VAL-155 | 6.17, 4.26 |
| (Z,E)-Farnesol | ASP-156 | 3.16 | TRP-205 | 5.81, 6.85 |
|  | - | - | MET-157 | 5.01 |
|  | - | - | CYS-203 | 4.15 |
|  | - | - | VAL-155 | 5.48 |
| 4-Methyl-1,5-Heptadiene | - | - | MET-157 | 4.07, 4.15 |
|  | - | - | TRP-205 | 6.87 |
| Hydroquinone | MET-157 | 3.95 | MET-157 | 4.36 |
|  | - | - | ASP-156 | 3.51 |
| trans-13-Docosenamide | ARG-160 | 5.21 | MET-157 | 4.11 |
|  | GLU-159 | 4.36 | - | - |
| Aspirin | GLY-154 | 3.71 | - | - |

**Table S7.** Molecular interactions with the best ligand efficiencies for compounds binding with TIRAP (PDB: 4FZ5).

| **Ligands** | **Hydrogen Bond Interactions** | | **Hydrophobic Bond Interactions** | |
| --- | --- | --- | --- | --- |
|  | **Amino Acid Residue** | **Distance (Å)** | **Amino Acid Residue** | **Distance (Å)** |
| 3-Furaldehyde | LEU-107 | 5.08 | LYS-210 | 4.44 |
|  | VAL-88 | 4.21 | - | - |
| 4-Ethylresorcinol | SER-110 | 3.87 | LYS-210 | 4.24, 4.72 |
|  | GLY-109 | 4.49 | - | - |
|  | TYR-106 | 3.71, 4.89 | - | - |
| Catechol | LEU-107 | 4.87 | LEU-107 | 5.06 |
|  | ASP-87 | 4.29 | VAL-213 | 6.30 |
|  | - | - | VAL-88 | 6.55 |
| Methylcyclohexane | - | - | VAL-213 | 4.40 |
|  | - | - | LEU-217 | 4.94 |
|  | - | - | LEU-107 | 5.13 |
| Hydroquinone | TYR-106 | 4.57 | LYS-210 | 4.86 |
| Phenol | LEU-107 | 4.61 | LEU-107 | 4.90 |
|  | - | - | VAL-88 | 7.09 |
|  | - | - | LEU-217 | 7.05 |
|  | - | - | VAL-213 | 5.77 |
|  | - | - | LYS-210 | 4.68 |
| Aspirin | LYS-210 | 4.51 | - | - |
|  | LEU-107 | 5.10 | - | - |

**Table S8.** Molecular interactions with the best ligand efficiencies for compounds binding with TLR4 (PDB: 3FXI).

| **Ligands** | **Hydrogen Bond Interactions** | | **Hydrophobic Bond Interactions** | |
| --- | --- | --- | --- | --- |
|  | **Amino Acid Residue** | **Distance (Å)** | **Amino Acid Residue** | **Distance (Å)** |
| Coniferol | LYS-125 | 5.26 | TYR-131 | 6.95 |
|  | GLU-439 | 4.18 | VAL-82 | 6.10 |
|  | ARG-90 | 4.87 | PHE-126 | 5.42 |
|  | - | - | SER-441 | 3.87 |
| 4-Methyl-1,5-Heptadiene | - | - | TYR-131 | 5.67 |
|  | - | - | VAL-82 | 5.07 |
|  | - | - | ILE-124 | 5.68 |
|  | - | - | PHE-126 | 5.42 |
|  | - | - | LEU-87 | 6.08 |
| Catechol | SER-441 | 3.06 | SER-441 | 4.27, 3.38 |
|  | LYS-126 | 5.35 | VAL-82 | 6.44 |
| Hydroquinone | LYS-125 | 5.29 | SER-441 | 3.83 |
|  | - | - | PHE-126 | 5.46 |
|  | - | - | VAL-82 | 6.17 |
| Phenol | SER-441 | 3.12 | VAL-82 | 5.13 |
|  | - | - | SER-441 | 4.10 |
|  | - | - | PHE-126 | 5.88 |
| Sulcatone | SER-441 | 3.36 | ILE-124 | 5.18 |
|  | - | - | ILE-80 | 4.99 |
|  | - | - | PHE-126 | 5.61 |
|  | - | - | LEU-87 | 5.61 |
| Aspirin | ARG-90 | 4.71 | ARG-90 | 4.80 |
|  | GLU-439 | 3.67 | - | - |

**Table S9.** Molecular interactions with the best ligand efficiencies for compounds binding with TRAF6 (PDB: 3HCT).

| **Ligands** | **Hydrogen Bond Interactions** | | **Hydrophobic Bond Interactions** | |
| --- | --- | --- | --- | --- |
|  | **Amino Acid Residue** | **Distance (Å)** | **Amino Acid Residue** | **Distance (Å)** |
| Coniferol | ARG-6 | 3.53 | LYS-96 | 7.04, 4.55 |
| 2-(2-Hydroxy-2-phenylethyl)-3,5,6-trimethylpyrazine | GLN-54 | 3.90 | ARG-6 | 3.84, 5.00 |
|  | ARG-6 | 3.84 | PRO-5 | 4.54 |
|  | - | - | ACE-53 | 4.09 |
|  | - | - | PRO-63 | 7.05 |
|  | - | - | ARG-7 | 6.53 |
| 3-Furaldehyde | LYS-96 | 4.72 | ARG-7 | 4.04, 4.04 |
|  | GLN-54 | 5.03 | PRO-63 | 4.72 |
|  | - | - | ILE-73 | 4.41 |
| Coumaran | - | - | LYS-96 | 5.80, 4.67 |
|  | - | - | PRO-63 | 5.78, 5.73 |
|  | - | - | TYR-62 | 6/06 |
|  | - | - | MET-64 | 5.98 |
|  | - | - | GLU-61 | 5.07 |
| Phenol | LYS-96 | 5.22 | PRO-63 | 6.68 |
| Sulcatone | CYS-93 | 4.08 | ARG-7 | 5.40 |
|  | LYS-96 | 4.32 | ARG-6 | 4.57 |
|  | ILE-72 | 4.57 | - | - |
|  | MET-64 | 5.22 | - | - |
|  | PRO-63 | 5.29 | - | - |
| Aspirin | LYS-96 | 6.20 | PRO-63 | 6.97 |
|  | GLN-54 | 4.77, 3.93 | - | - |
|  | ARG-7 | 5.63 | - | - |
|  | ARG-6 | 4.57 | - | - |


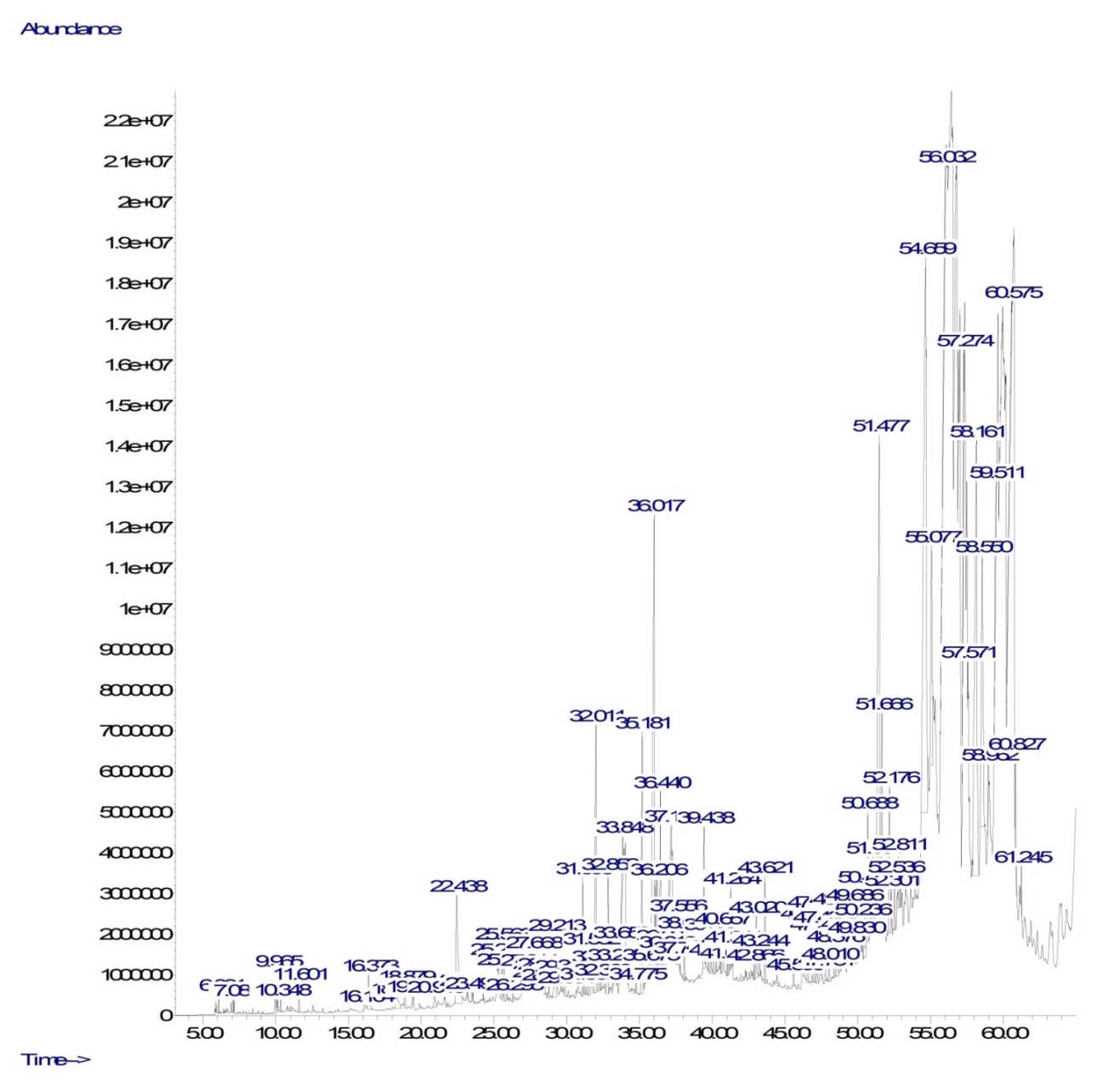


Figure S1. GC-MS chromatograph of the methanolic extract of *Argyreia capitiformis* stem.


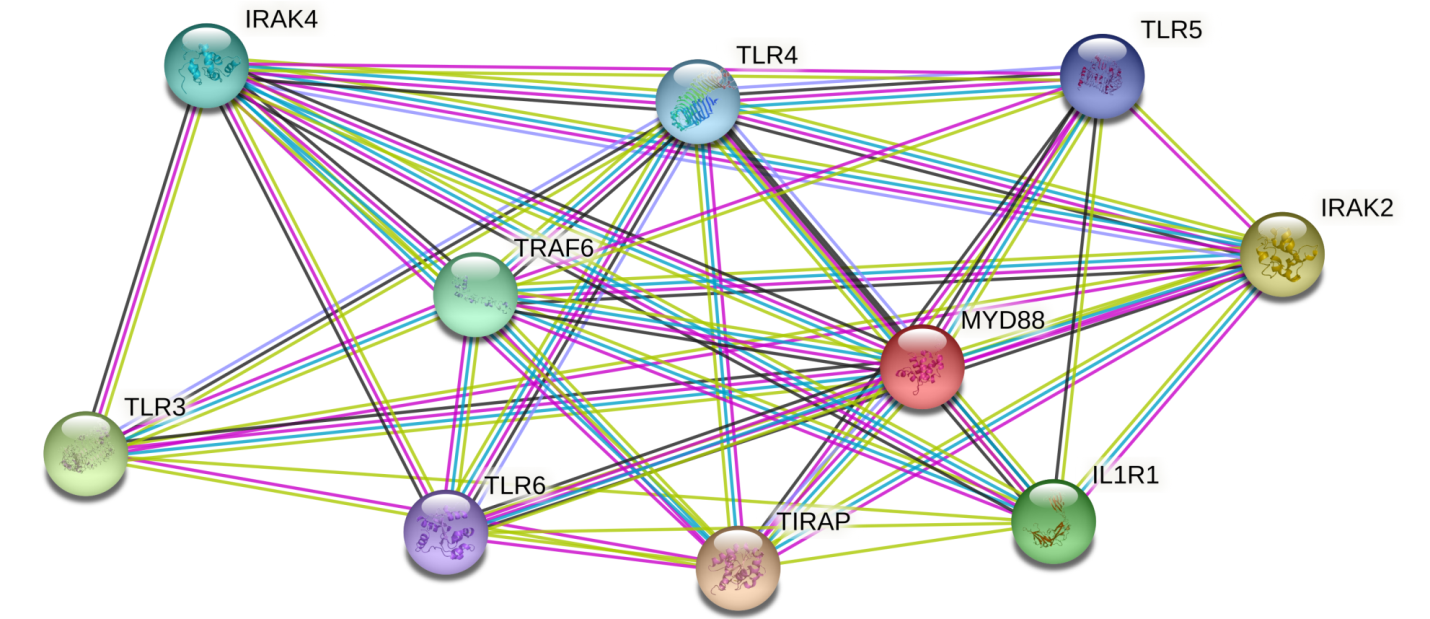


Figure S2. Protein-protein interaction (PPI) network of 10 proteins.


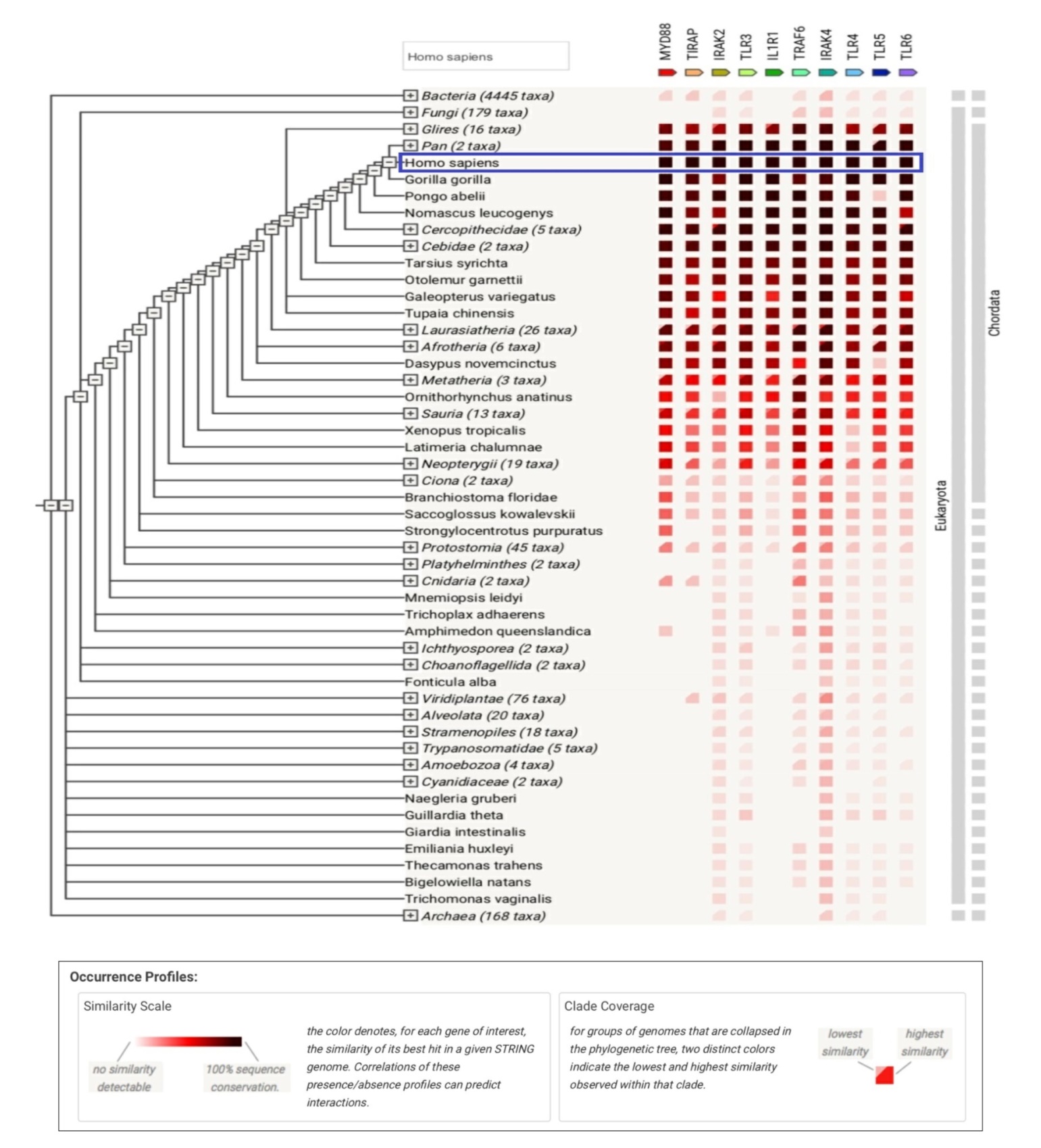


Figure S3. Gene co-occurrence of 10 proteins for *Homo sapiens.*


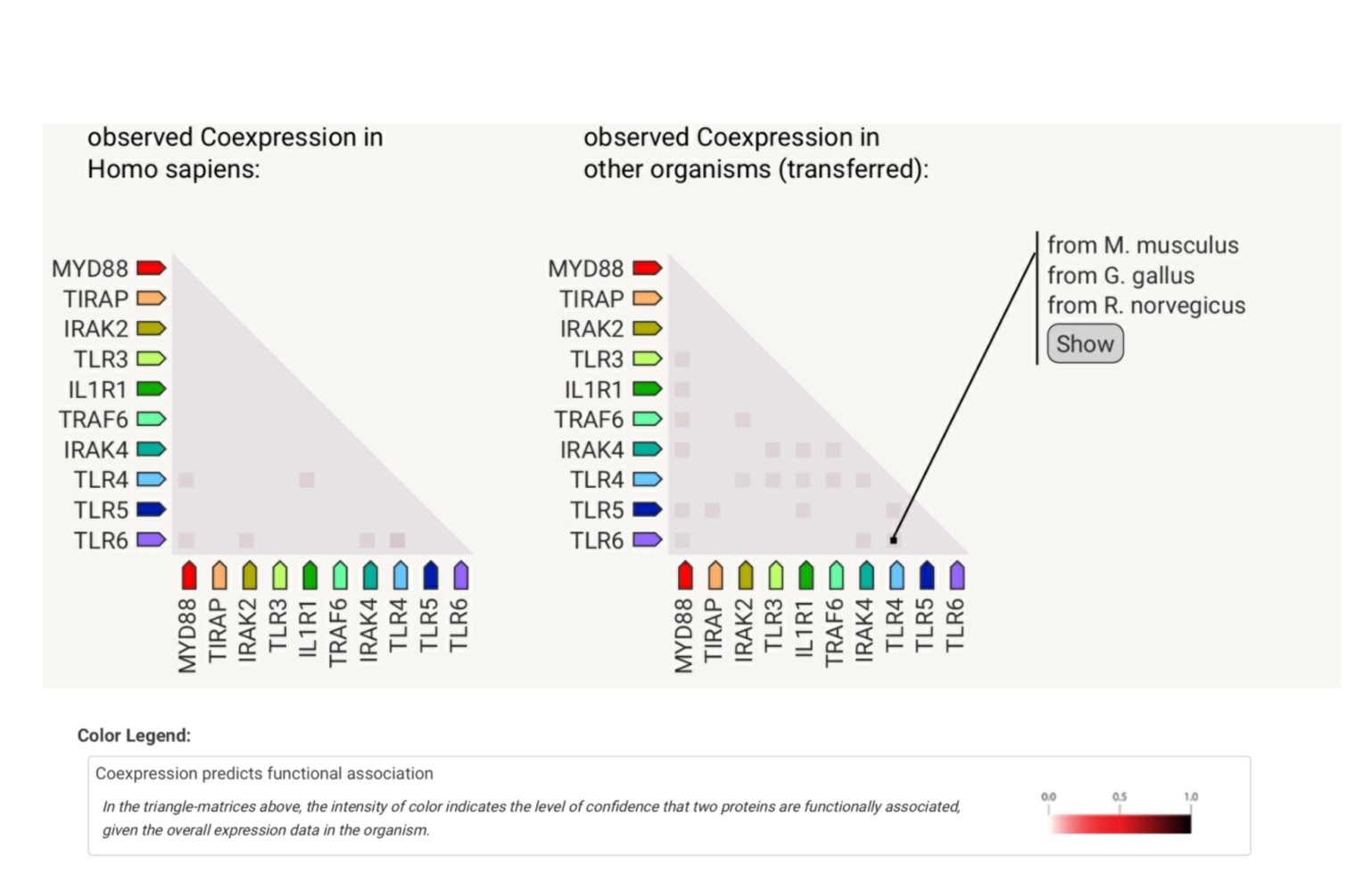


Figure S4. Gene coexpression of 10 proteins in PPI network.


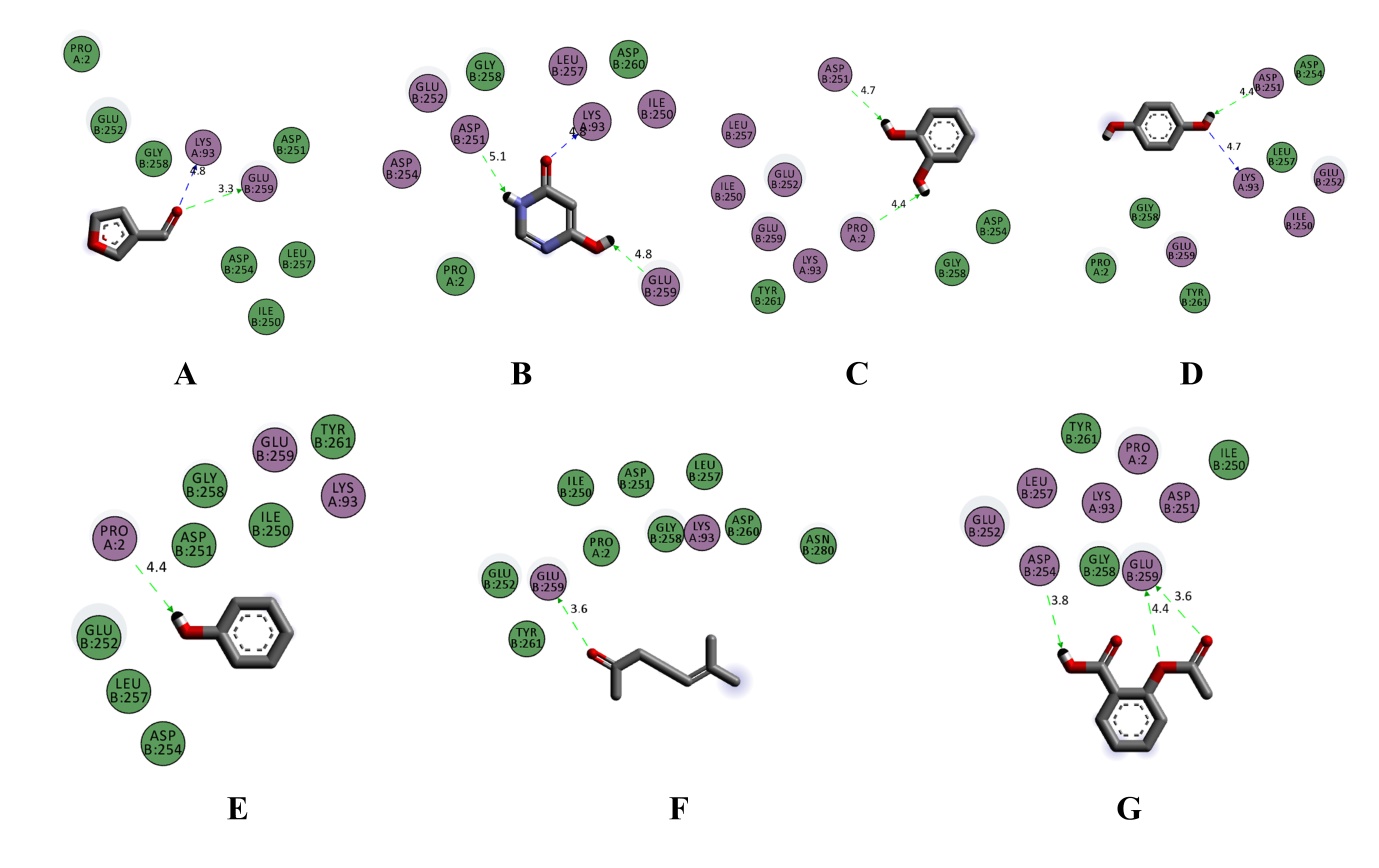


**Figure S5.** Two-dimensional representations of the best ligand efficiencies for compounds binding with IL1R1 (PDB: 1ITB). 3-furaldehyde (A), 4(1H)-pyrimidinone, 6-hydroxy- (B), catechol (C), hydroquinone (D), phenol (E), sulcatone (F), and aspirin (G) are shown.


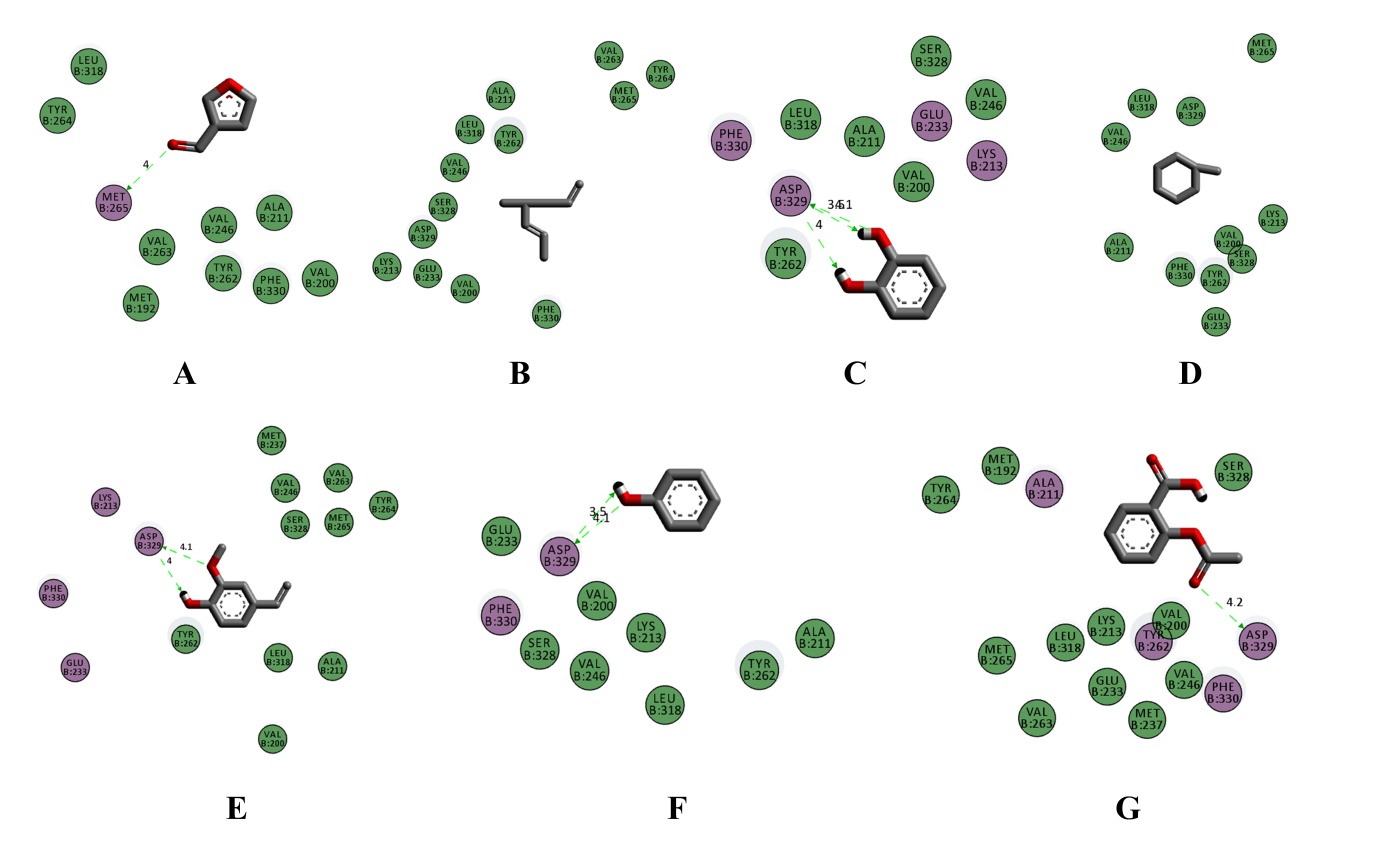


Figure S6. Two-dimensional representations of the best ligand efficiencies for compounds binding to IRAK4 (PDB: 6EGA). 3-furaldehyde (A), 4-methyl-1,5-heptadiene (B), catechol (C), methylcyclohexane (D), p-vinylguaiacol (E), phenol (F), and aspirin (G) are shown.


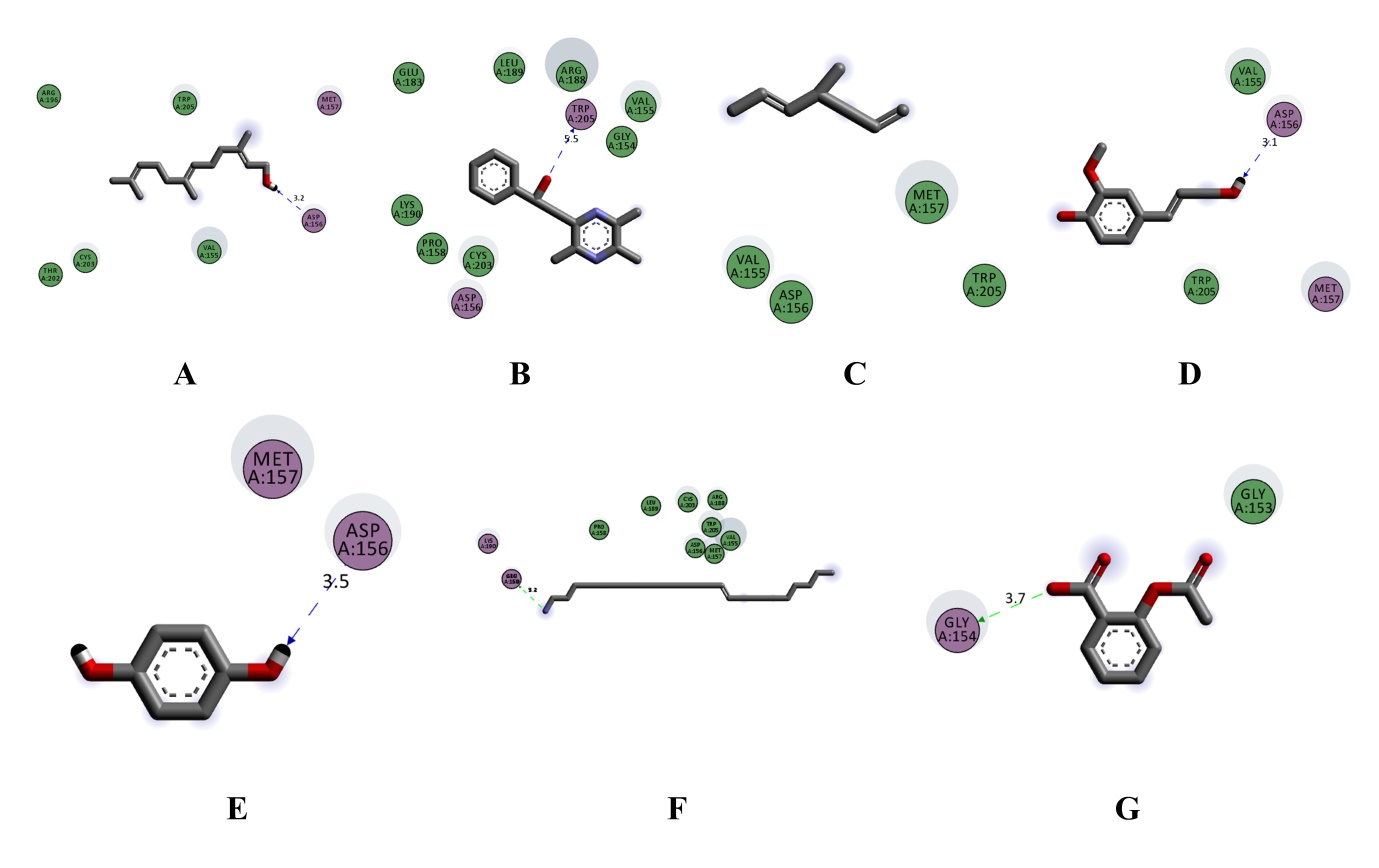


Figure S7. Two-dimensional representations of best the ligand efficiencies for compounds binding to MYD88 (4EO7). (Z,E)-farnesol (A), 2-(2-hydroxy-2-phenylethyl)-3,5,6-trimethylpyrazine (B), 4-methyl-1,5-heptadiene (C), coniferol (D), hydroquinone (E), trans-13-docosenamide (F), and aspirin (G) are shown.


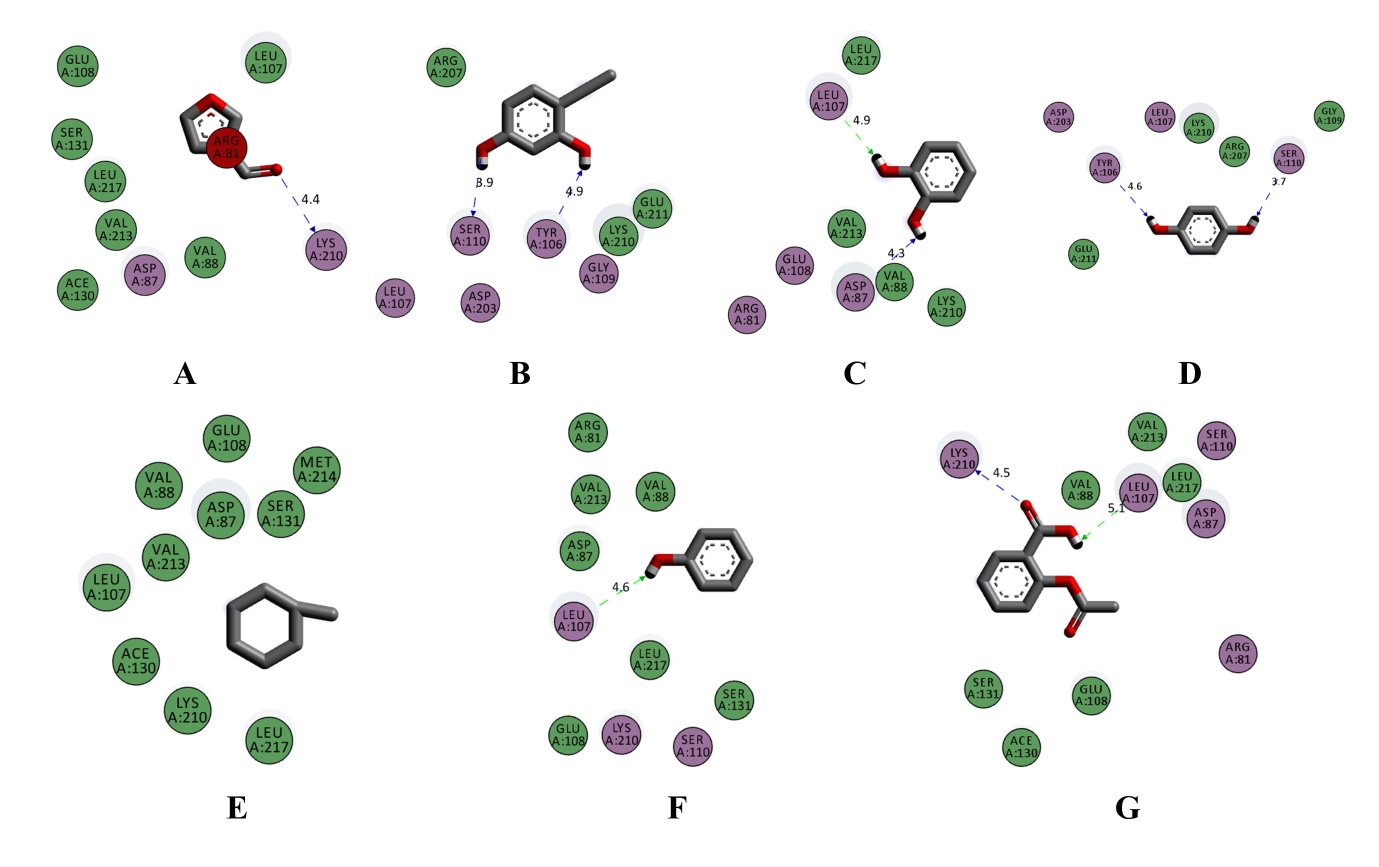


Figure S8. Two-dimensional representations of the best ligand efficiencies for compounds binding to TIRAP (PDB: 4FZ5). 3-furaldehyde (A), 4-ethylresorcinol (B), catechol (C), hydroquinone (D), methylcyclohexane (E), phenol (F), and aspirin (G) are shown.


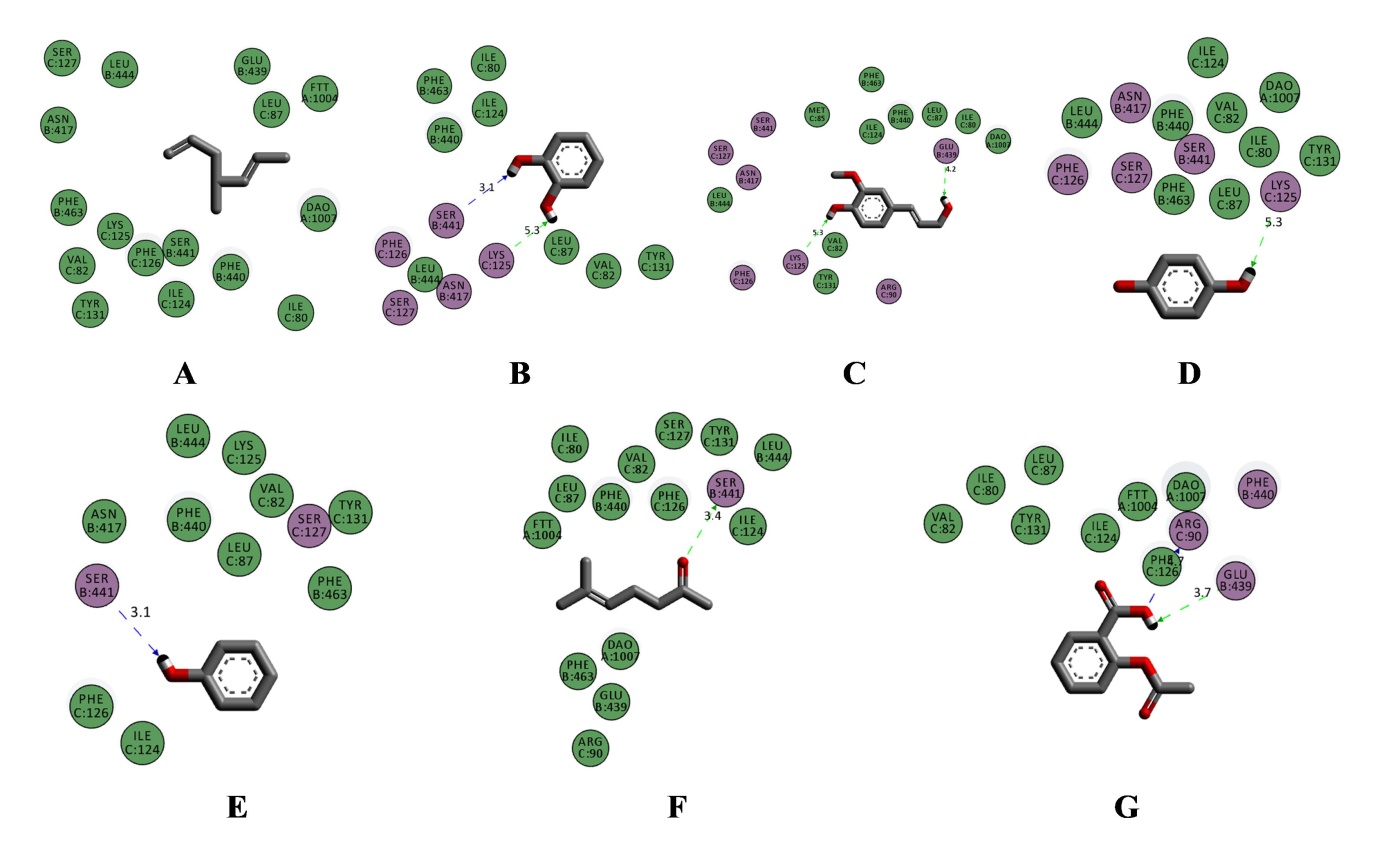


Figure S9. Two-dimensional representations of best ligand efficiencies for compounds against TLR4 (PDB: 3FXI). 4-methyl-1,5-heptadiene (A), catechol (B), coniferol (C), hydroquinone (D), phenol (E), sulcatone (F), and aspirin (G) are shown.


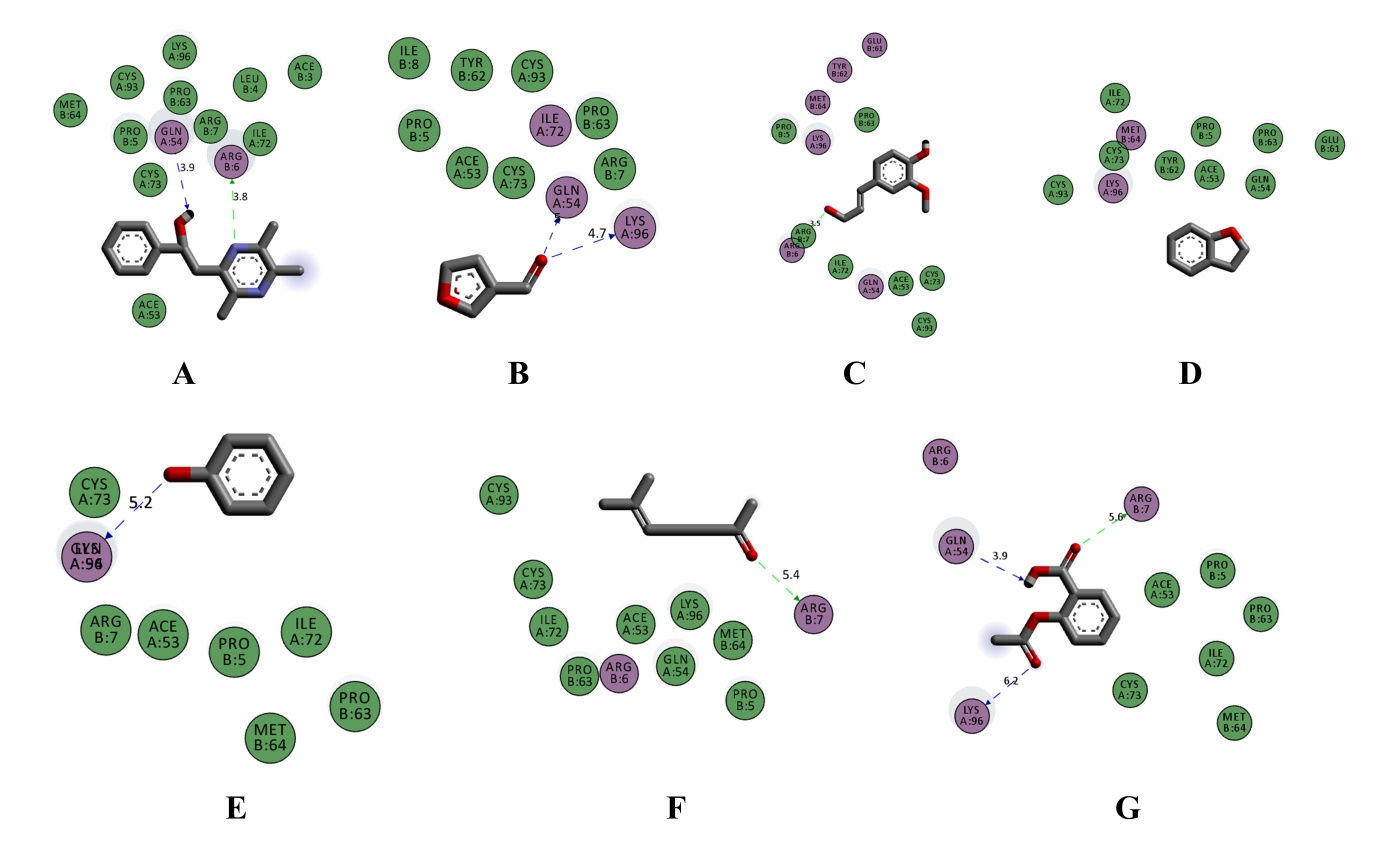


Figure S10. Two-dimensional representations of the best ligand efficiencies for compounds against TRAF6 (PDB: 3HCT). 2-(2-Hydroxy-2-phenylethyl)-3,5,6-trimethylpyrazine (A), 3-furaldehyde (B), coniferol (C), coumaran (D), phenol (E), sulcatone (F), and aspirin (G) are shown.
